# The deubiquitinating enzyme USP44 suppresses hepatocellular carcinoma progression by inhibiting Hedgehog signaling and PDL1 expression

**DOI:** 10.1038/s41419-023-06358-y

**Published:** 2023-12-14

**Authors:** Sisi Chen, Binghai Zhou, Wei Huang, Qing Li, Ye Yu, Xiuqing Kuang, Huabin Huang, Wei Wang, Peiyi Xie

**Affiliations:** 1 Department of Neurology, Second Affiliated Hospital of Nanchang University, Nanchang, 330006 Jiangxi PR China; 2 Hepato-Biliary-Pancreatic Surgery Division, Department of General Surgery, Second Affiliated Hospital of Nanchang University, Nanchang, 330006 Jiangxi PR China; 3 Department of Liver Surgery and Transplantation, Liver Cancer Institute, Zhongshan Hospital, Fudan University, Key Laboratory of Carcinogenesis and Cancer Invasion, Ministry of Education, Shanghai, 200032 PR China; 4 Department of Pathology, Second Affiliated Hospital of Nanchang University, Nanchang, 330006 Jiangxi PR China; 5 Department of Physical Examination, Second Affiliated Hospital of Nanchang University, Nanchang, 330006 Jiangxi PR China; 6 Department of Medical Imaging, Second Affiliated Hospital of Nanchang University, Nanchang, 330006 Jiangxi PR China

**Keywords:** Liver cancer, Cell biology

## Abstract

Hepatocellular carcinoma (HCC) is one of the deadliest malignancies in the world. Research into the key genes that maintain the malignant behavior of cancer cells is crucial for the treatment of HCC. Here, we identified ubiquitin‐specific peptidase 44 (USP44), a member of the deubiquitinase family, as a novel regulator of HCC progression. The tumor suppressive function of USP44 was evaluated in a series of in vitro and in vivo experiments. Through quantitative proteomics examination, we demonstrated that USP44 inhibits HCC PDL1 expression by downregulating the Hedgehog (Hh) signaling pathway. Mechanistically, we found that USP44 directly interacts with Itch, an E3 ligase involved in Hh signaling, and promotes the deubiquitination and stabilization of Itch. These events result in the proteasomal degradation of Gli1 and subsequent inactivation of Hh signaling, which ultimately suppresses PDL1 expression and the progression of HCC. Furthermore, the HCC tissue microarray was analyzed by immunohistochemistry to evaluate the pathological relevance of the USP44/Itch/Gli1/PDL1 axis. Finally, the Gli1 inhibitor GANT61 was found to act in synergy with anti-PDL1 therapy. Overall, USP44 can act as a suppressive gene in HCC by modulating Hh signaling, and co-inhibition of Gli1 and PDL1 might be an effective novel combination strategy for treating HCC patients.

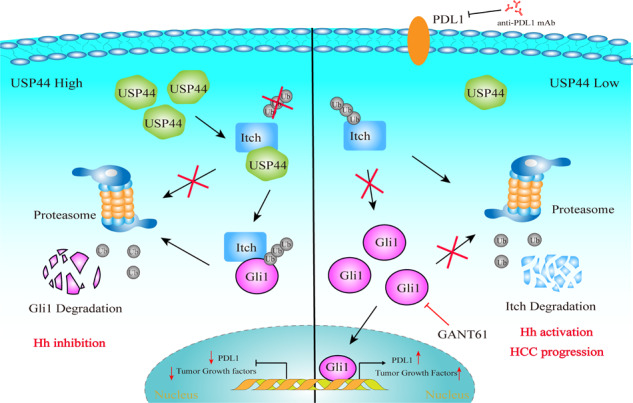

## Introduction

With an estimated 906,000 new cases and 830,000 deaths, hepatocellular carcinoma (HCC) is the third most lethal malignancy worldwide [1]. Rapid proliferation and distant metastasis remain the major causes contributing to the poor prognosis of HCC patients. Although great breakthroughs have been made in the evolution of surgical removal, chemotherapy, radiofrequency ablation, transarterial chemoembolization, tyrosine kinase inhibitors or immune checkpoint inhibitors such as anti-programmed cell death (/ligand) 1 (PD1/PDL1) immunotherapy, the survival outcomes of advanced HCC patients are still frustrating [2–4]. Thus, it is imperative to explore the underlying molecular mechanisms of HCC progression and identify effective strategies for HCC treatment.

Deubiquitinating enzymes (DUBs) are proteolytic enzymes that can reverse ubiquitination-mediated protein degradation through cleaving ubiquitin from substrate proteins [5]. Deregulation of DUBs can result in significant physiological consequences and is correlated with multiple diseases including inflammatory diseases [6], neural degeneration [7] and cancer [8]. The deubiquitinating enzyme ubiquitin‐specific peptidase 44 (USP44), a member of the ubiquitin-specific protease family, is located at 12q22 and encodes an 82-kD protein [9]. Recently, USP44 was found to be involved in the progression of multiple malignancies [9–11]. However, the role and underlying mechanism of USP44 in HCC remain elusive.

The Hedgehog (Hh) signaling pathway is crucial for regulating normal cell proliferation and embryonic development [12, 13]. Its aberrant activation can result in the onset and development of multiple human cancers, including HCC. Hh signaling can be initiated through the interaction of Hh ligands with the Patched1 (Ptch1) receptor, which relieves its repressive effect on the coreceptor Smoothened (SMO). This interaction triggers the activation of the glioma-associated oncogene homolog (Gli) family upon dissociation from the suppressor of fused (SuFu) protein, a negative modulator of the signaling pathway. In mammals, three members are identified in the Gli family, including Gli1, Gli2, and Gli3, among which Gli1 is the only transcriptional activator [14, 15]. Activated Gli1 triggers the transcription of a panel of genes, including Bcl2, c-Myc, Nanog, FOXS1, and PDL1, and promotes the proliferation, survival, invasion, and migration of cancer cells [16–20]. Accordingly, targeting the Hh/Gli1 pathway is expected to be a promising strategy for cancer therapy. Gli1 is regulated by several types of posttranslational modifications (PTMs), including ubiquitination [21]. Itch has emerged as an E3 ubiquitin ligase that promotes the ubiquitination and degradation of the Gli1 protein [22]. However, the upstream signaling and biological processes that control Itch expression are poorly understood.

In the present study, we revealed a previously undescribed role of USP44 in suppressing HCC progression and delineated that the USP44/Itch/Gli1 axis is critical for triggering the progression and PDL1 expression of HCC. Notably, targeting the USP44/Itch/Gli1 axis using the Gli1 inhibitor GANT61 acts in synergy with anti-PDL1 therapy, which may benefit patients with HCC.

## Materials and methods

### Patients and samples

The study included two tissue microarrays (TMA1 and TMA2) derived from patients who underwent curative resection in the Second Affiliated Hospital of Nanchang University. TMA1 is composed of 191 HCC samples and TMA2 is composed of 56 HCC samples. To measure the mRNA and protein expression of USP44 in tissue from HCC patients, specimens were randomly obtained from 56 HCC patients who underwent curative resection at the Second Affiliated Hospital of Nanchang University. To detect the protein expression of USP44, Itch, Gli1, and PDL1 in tissue from HCC, specimens were randomly obtained from another cohort of 25 HCC patients who underwent curative resection. All patients signed informed consent forms approved by the Ethics Committee of Second Affiliated Hospital of Nanchang University.

### Immunofluorescence

HCC cells were fixed with 4% paraformaldehyde for 10 min at room temperature. Then, fixed HCC cells were incubated in 5% Triton X-100 for 10 min and blocked with 5% bovine serum (BSA) (Sigma–Aldrich, St. Louis, MO, USA), followed by incubation with primary antibodies overnight at 4 °C. After washing with PBS, anti-mouse Alexa Fluor 488 or anti-rabbit 546 dye conjugates were used as secondary antibodies (Life Technologies, Carlsbad, CA). ProLong Gold antifade reagent with DAPI (Invitrogen, Carlsbad, CA, USA) was used to stain nuclei. The localization of the proteins was viewed through a confocal laser scanning microscope (SP-II; Leica Microsystems, Wetzlar, Germany).

### EdU assay

For the 5-Ethynyl-20-deoxyuridine assay (EdU), 5 × 10^3^ cells were seeded in per well of 96-well plates. After the cells were cultured for 24 h, 5-ethynyl-20-deoxyuridine (EdU; RiboBio) was utilized to incubate the cells for 4 h. Then, the cells were incubated with 1x Apollo reaction cocktail for 30 min and the DNA contents of the cells were stained with Hoechst 33342 (5 mg/mL) in each well for 20 min. A fluorescence microscope was used to image the cells. To evaluate the degree of the correlation of mitotic index, the S-phase fraction was used as marker of cell proliferation as previously described [23].

### Cell Counting Kit-8 assay

Transfected cells (5 × 10^3^ per well) were seeded into 96-well plates. A CCK-8 assay was used to detect cell viability after 24, 48, 72, 96, 120 and 144 h. 10 μL of CCK-8 reagent (Dojindo Laboratories, Kumamoto, Japan) was added to each well. After incubation for 1 h in cell incubator, the absorbance at wavelength of 450 nm was recorded.

### Wound-healing migration assay

To detect the migration ability, transfected cells were plated into 6-well plates. When cells grew to 80% to 90% confluence, a scratched cell-free area was made through a 200 μl pipette tip. Subsequently, the cells were incubated, and wound closure was observed at 0 h, 24 h and 48 h. Three randomly selected wound areas were analyzed. The wound-healing rate was measured as 100%-(the ratio of the remaining cell-free area to the area of the initial wound) (estimated as a mean percentage) by Image J software [24].

### Transwell Matrigel invasion assays

For the invasion assay, a Transwell system (Millcell, Germany) with Matrigel (BD Biosciences) coated above the membrane was employed. The indicated stably transfected cells were suspended in serum-free DMEM at a concentration of 2 × 10^4^/ml. Then, 500 µL of cell suspension was loaded in the upper chamber. The bottom chamber was filled with 500 µL of DMEM supplemented with 10% FBS. 24 h later, the migratory and invasive cells were fixed with 4% paraformaldehyde for 20 min at time temperature. Then, 0.1% crystal violet was employed to stain the cells for 15 min. Cells in five randomly selected fields of view were counted. Relative cell invasion ratio refers to the relative ratio of invasive cell number between different groups [24].

### Animal studies

All in vivo animal-related experiments were approved by the Ethics Committee for Animal Experiments of the Second Affiliated Hospital of Nanchang University (Nanchang, China). BALB/C nude mice and wild-type C57BL/6 mice (5–6 weeks, male) purchased from Charles River Laboratory (Beijing, China) were maintained in a temperature-controlled, specific-pathogen-free (SPF) animal laboratory.

USP44^fl/fl^ and Alb-cre mice (both C57BL/6) were obtained from Shanghai Model Organisms Center (Shanghai, China) and bred in a pathogen-free environment according to the guidelines of the animal facility in Nanchang University. USP44^fl/fl^ mice were crossed with Alb-cre mice to generate liver conditional USP44-knockout (USP44 cKO) mice. Seven-week-old male USP44 cKO mice were used for subsequent experiments.

For the subcutaneous tumor model, mice were randomly divided into different subgroups (6 mice per group) and were subcutaneously injected with 3 × 10^6^ control and USP44 stably overexpressing HCC cells tumor cells. Mice were sacrificed 4 weeks after inoculation of tumor cells, and the tumor weights were measured. For metastatic tumor models, HCC cells (1 × 10^7^) were inoculated into the caudal vein of mice. An in vivo imaging system (IVIS, PerkinElmer, USA) was used to monitor tumor progression in mice (5 mice per group). After the mice were sacrificed, lung tissues of these mice were collected and haematoxylin-eosin (H&E) staining was employed to evaluate pulmonary metastatic nodules.

For the orthotopic tumor model, after the subcutaneous tumors grew to 1 cm^3^, subcutaneous tumor tissues were resected and cut into 1-mm^3^ pieces for orthotopic xenograft implantation. Xenografted mice were sacrificed after 7 weeks and the volume of tumors was measured using the formula: V = [length/2] × [width^2^].

For combination therapy, C57BL/6 tumor-bearing mice were injected intraperitoneally with 50 mg/kg GANT61 (Selleck) and/or 100 μg anti-PDL1 mAb (BioXCell) or isotype control mAb. An ultrasound imaging platform was used to evaluate tumor growth every 5 days.

### Statistical analysis

All statistical analyses were conducted using GraphPad Prism 6.0 software. Student’s *t*-test was used for two-group comparisons and one-way ANOVA was used for multiple comparisons. Differences were considered statistically significant when *P* < 0.05. The Kaplan–Meier method was utilized to draw survival curves. Spearman’s correlation test was employed in correlation analyses. All experiments were conducted at least 3 times and all results are shown as the mean ± SD.

(Additional materials and methods can be obtained from the supplementary materials and methods.)

## Results

### USP44 expression is decreased in HCC and correlates with a poor prognosis

**Fig. 1:**
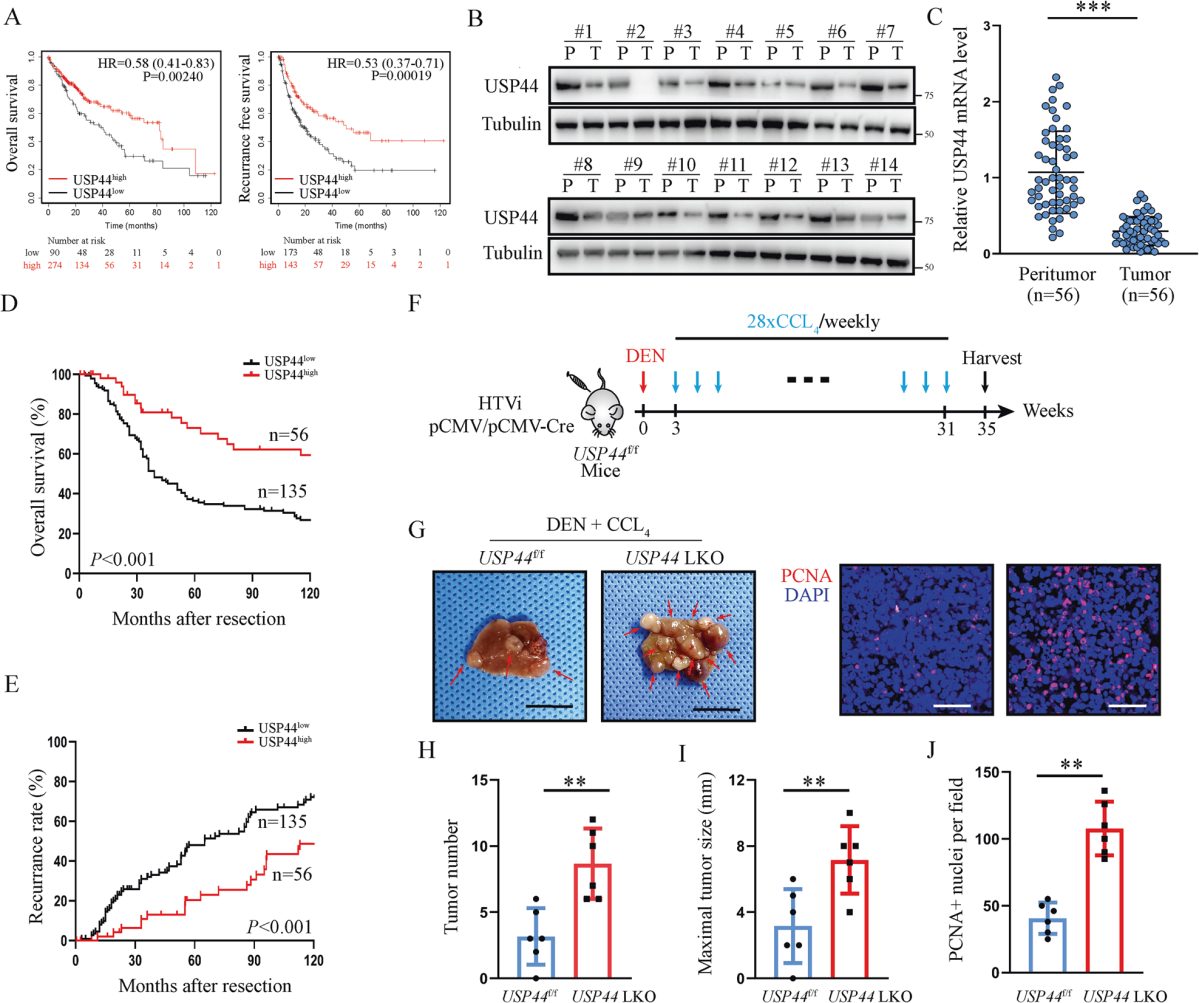
USP44 is downregulated and associated with a poor prognosis in HCC. **A** Kaplan–Meier analysis of the overall survival and recurrence-free survival of HCC patients from the TCGA database. **B** Representative images of USP44 expression in paired tumors and peritumoral tissues from 14 patients with HCC using western blot. **C** mRNA expression of USP44 in 56-paired HCC tissues. **D**, **E** Low expression of USP44 was significantly related to poor overall survival and high recurrence rate in HCC patients. **F** Illustration of the mouse model of DEN/CCl_4_-induced hepatocarcinogenesis in USP44 flox/flox conditional knockout (KO) mice and their USP44 flox/flox littermates. **G** Gross appearance of representative livers with tumors were obtained from the indicated groups and PCNA staining of the tumor tissues. Scale bar, 1 cm (left panel) and 50 μm (right panel). **H**, **I** Tumor number and maximal tumor size were calculated in the spontaneous tumor models. **J** The relative number of PCNA^+^ cells were counted. *p < 0.05, **p < 0.01, ***p < 0.001 as indicated.

Using The Cancer Genome Atlas (TCGA) database, we found that low expression of USP44 is significantly correlated with poor prognosis in HCC patients (Fig. 1A). However, to the best of our knowledge, the role of USP44 in HCC has seldomly been reported. To evaluate the potential role of USP44 in HCC, we first evaluated the protein and mRNA levels in our in-house 56-paired HCC samples. We found that USP44 expression was significantly decreased in HCC compared with peritumor tissues (Fig. 1B, C). Next, we performed immunohistochemistry (IHC) using tissue microarrays (TMAs) from 191 HCC patients who underwent curative resection and we found that the median overall survival (OS) time was significantly shorter in patients with low USP44 expression than in those with high expression (Fig. 1D), which is consistent with the data from the TCGA database. Additionally, HCC patients with low USP44 expression also had a higher recurrence rate (Fig. 1E). Furthermore, we found that patients with low USP44 expression were prone to larger tumor size, distant metastasis, and late-stage disease (Table S1). Additionally, multivariate analysis showed that low USP44 was an independent predictor of both OS and recurrence-free survival (RFS) (Table S2). Next, we demonstrated that USP44 knockout in murine hepatocytes impaired the tumor suppressive function of USP44 in a mouse model of diethylnitrosamine (DEN)/carbon tetrachloride (CCl_4_)-induced hepatocarcinogenesis (Fig. 1F–I). We confirmed that USP44 deletion significantly promoted the proliferation of tumor cells through immunofluorescence of proliferating cell nuclear antigen (PCNA) in tumor samples (Fig. 1G, J). Above all, our data suggest that USP44 may have a tumor suppressive role in HCC and has the potential to serve as a diagnostic biomarker for HCC.

### USP44 inhibits HCC proliferation and metastasis in vitro and in vivo

**Fig. 2:**
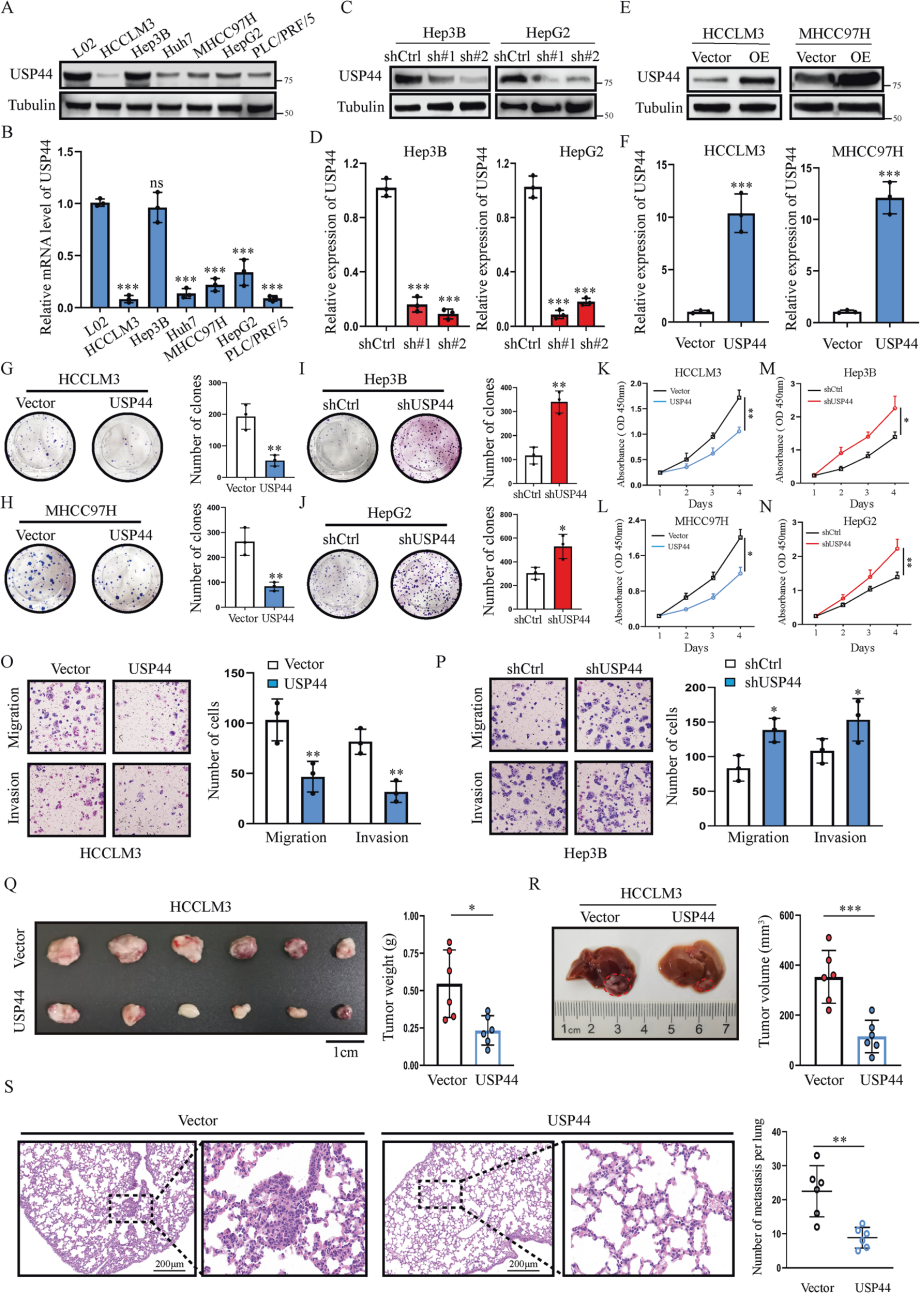
USP44 inhibits HCC metastasis and progression in vitro and in vivo. **A**, **B** USP44 expression in different HCC cell lines and normal liver cell line L02 examined by western blot and qRT-PCR. **C**–**F** The outcomes of USP44 knockdown and overexpression were examined by western blot and qRT-PCR in HCC cells. **G**–**N** The Colony formation assay and the CCK-8 assay were used to detect the effect of USP44 on HCC proliferation. **O**, **P** The transwell assay was used to detect the effect of USP44 on the migration and invasion activities of HCC cells. **Q**, **R** Subcutaneous tumor model and orthotopic xenograft models were constructed using indicated cells, and tumor weights were measured at the endpoint (*n* = 6). **S** Representative H&E staining images of the lungs (left) and the number of metastases per lung (right) in each group. Data are obtained from three independent biological replicates and are presented as mean ± SD. Scale bar, 200 μm. *p < 0.05, **p < 0.01, ***p < 0.001 as indicated.

To demonstrate the tumor suppressive role of USP44 in HCC, we further performed in vitro and in vivo experiments. We first detected the endogenous USP44 levels in different HCC cell lines or the control nontumoral cell line L02 using western blot and quantitative RT-PCR (qRT-PCR) (Fig. 2A, B). To further investigate the role of USP44 in HCC cell lines, we used USP44 short hairpin RNAs (shRNAs) to construct USP44-knockdown Hep3B and HepG2 cell lines (Fig. 2C, D). We also used USP44-overexpressing plasmids to establish USP44-overexpressing HCCLM3 and MHCC97H cell lines (Fig. 2E, F). The expression levels of USP44 in these cells with USP44 knockdown or overexpression were confirmed by both western blot and qRT-PCR (Fig. 2C–F). Using colony formation and Cell Counting Kit-8 (CCK-8) assays, we found that USP44 overexpression suppressed HCC cell growth, while USP44 knockdown showed the opposite effects (Fig. 2G–N). Furthermore, using Transwell assays, we found that USP44 overexpression significantly decreased the ability of HCC cells to migrate and invade, whereas USP44 downregulation exerted the opposite effect (Fig. 2O, P). To explore the role of USP44 in HCC growth in vivo, we established a subcutaneous tumor model and a liver orthotopic xenograft tumor model in mice. Consistently, we found that USP44 upregulation effectively inhibited tumor growth in mice (Fig. 2Q, R). Additionally, tumors with USP44 overexpression had less lung metastasis than the control group (Fig. 2S). Collectively, these results suggest the tumor suppressive role of USP44 in HCC.

### USP44 inactivates Hh signaling and PDL1 expression through Itch

**Fig. 3:**
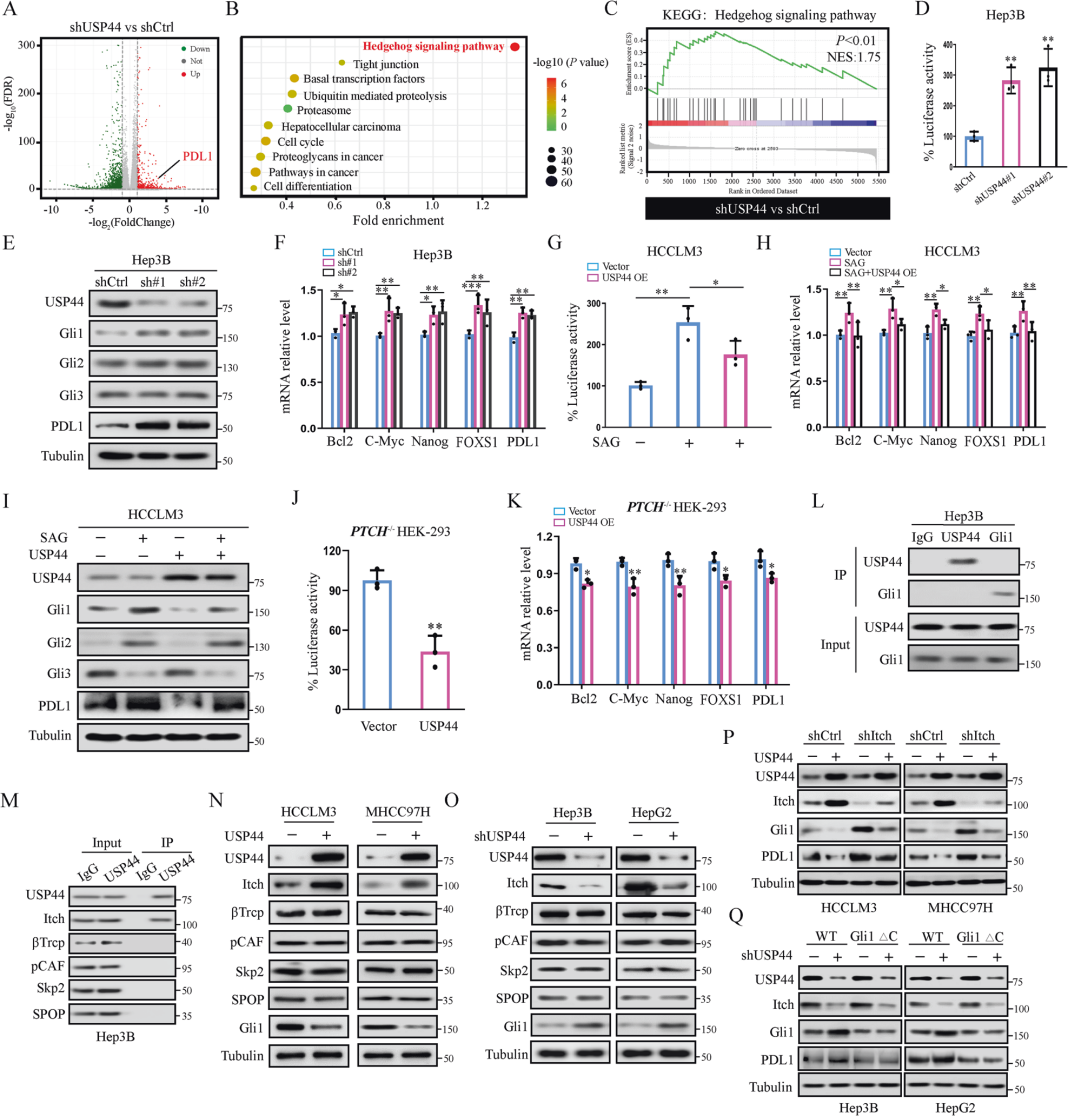
USP44 overexpression inhibits Hh signaling and PDL1 expression through Itch. **A** Volcano plots showing DEPs in the shUSP44 group compared with the control group in Hep3B cells. |log2FC | >1, *p*-value < 0.05. **B**, **C** KEGG pathway enrichment and gene set enrichment analysis (GSEA) results indicated that hyperactivation of Hh signaling was observed in shUSP44 HCC cells compared with the control group. **D** Luciferase activity of the Hh pathway-reporter in shCtrl or shUSP44 transfected Hep3B cells. **E** Western blot for protein expression of Gli1, Gli2, Gli3, and PDL1 in shCtrl or shUSP44 transfected HCC cells. **F** mRNA level of Hh signaling target genes in shCtrl or shUSP44 transfected Hep3B cells. **G** Luciferase activity of the Hh pathway-reporter in control vector or exogenous USP44-overexpressing HCCLM3 cells treated with or without SAG (200 nM for 24 h). **H** mRNA level of Hh signaling target genes in control vector or exogenous USP44-overexpressing HCCLM3 cells treated with or without SAG (200 nM for 24 h). **I** Western blot for protein expression of Gli1, Gli2, Gli3, and PDL1 in control vector or exogenous USP44-overexpressing HCCLM3 cells treated with or without SAG (200 nM for 24 h). **J** Luciferase activity of the Hh signaling pathway-reporter in Ptch^−/−^HEK-293 cells expressing control vector or exogenous USP44. **K** mRNA level of Hh signaling target genes in Ptch^−/−^HEK-293 cells expressing control vector or exogenous USP44. **L** Interaction between USP44 and Gli1 was detected by immunoprecipitation followed by western blot analysis with the indicated antibodies. **M** Immunoprecipitation was employed to test the interaction between USP44 and the ubiquitin E3 ligases of Gli1. **N**, **O** Immunoblot for protein expression of the ubiquitin E3 ligases of Gli1 in HCC cells with different USP44 expression patterns. **P** Western blot for protein expression of Gli1 in HCC cells expressing control vector or exogenous USP44, with or without shItch transfection. **Q** Western blot for protein expression of Itch and Gli1 in shCtrl or shUSP44 expressing HCC cells transfected with wild-type Gli1 (Flag-Gli1 WT) or the mutant Gli1 (Flag-Gli1 ∆C). Data are obtained from three independent biological replicates and are presented as mean ± SD. *p < 0.05, **p < 0.01, ***p < 0.001 as indicated.

The aforementioned results prompted us to perform Tandem Mass Tag™ Quantitative Proteomics examination to identify the potential regulatory mechanism of USP44 in HCC. In the USP44-knockdown Hep3B cell line, we identified 2137 differentially expressed proteins (DEPs) compared with the control group, and PDL1 was significantly upregulated (Fig. 3A). In addition, KEGG pathway enrichment analysis indicated that UPS44 silencing markedly activated the Hh signaling pathway (Fig. 3B, C). To further verify the role of USP44 in Hh signaling, we used USP44-knockdown HCC cell lines and found that low USP44 expression enhanced the activity of the Hh pathway-reporter (Fig. 3D, Supplementary Fig. 1A). Next, we evaluated the expression of Glis, the terminal and most potent effector of Hh signaling. The western blot results showed that the expression of Gli1, but not Gli2 or Gli3, was dramatically increased in USP44-downregulated HCC cells (Fig. 3E, Supplementary Fig. 1B). Notably, PDL1 expression was also significantly upregulated with USP44 downregulation (Fig. 3E, Supplementary Fig. 1B). We further investigated the regulatory effect of USP44 on Hh signaling by detecting the expression of its target genes including Bcl2, C-Myc, Nanog, FOXS1 and PDL1. As expected, USP44-knockdown increased the mRNA levels of these target genes (Fig. 3F, Supplementary Fig. 1C). These findings were further confirmed by experiments employing ectopic overexpression of USP44 and treatment with the Hh pathway agonist SAG, in which the SAG-induced increase in reporter activity was counteracted by USP44 overexpression (Fig. 3G, Supplementary Fig. 1D). Additionally, the increase in the mRNA level of these target genes caused by SAG treatment was completely rescued by USP44 overexpression (Fig. 3H, Supplementary Fig. 1E). Consistent with these data, USP44 overexpression could impaired the increase in Gli1 and PDL1 expression induced by SAG treatment (Fig. 3I, Supplementary Fig. 1F, G). Furthermore, we examined the reporter activity of Hh signaling and the expression of its target genes in Ptch1^−/−^HEK-293 cells wherein the Hh signaling pathway was constitutively active. Following upregulating the expression of USP44 in Ptch1^−/−^HEK-293 cells, we observed a remarkable reduction in the reporter activity of the Hh signaling pathway and the mRNA level of the target genes (Fig. 3J, K).

However, the qRT-PCR results demonstrated no significant difference in the mRNA level of Glis in the USP44 knockdown or overexpression groups (Supplementary Fig. 1H–K), implying that Gli1 was modulated by USP44 in a posttranslational manner. Since USP44 could not directly interact with Gli1 (Fig. 3L), we hypothesized that USP44 negatively regulates Gli1 expression by affecting ubiquitin E3 ligase(s), which are known to destabilize Gli1. Among the known E3 ligases, including βTrCP, Itch, pCAF, Skp2, and SPOP, we found that USP44 was associated with only Itch (Fig. 3M). More importantly, overexpression of USP44 resulted in an increase in Itch expression and a subsequent decline in Gli1 protein levels (Fig. 3N), whereas its genetic inhibition led to the opposite effect (Fig. 3O). Additionally, the modulation of USP44 had no effect on the expression of other E3 ligases for Gli1 degradation or on the protein levels of other Itch substrates including LATS1/2, smad7, RASSF5 and wbp2 (Fig. 3N, O, Supplementary Fig. 2A, B), suggesting that USP44 might inhibit Gli1 expression by upregulating the Itch protein. Furthermore, we found that USP44 overexpression dramatically inhibited the expression of Gli1 and PDL1, while Itch knockdown profoundly suppressed the USP44-induced decline in Gli1 and PDL1 expression (Fig. 3P). In contrast, the downregulation of USP44 enhanced the expression of Gli1 and PDL1, whereas the upregulation of Itch diminished the increase in Gli1 and PDL1 caused by USP44 knockdown (Supplementary Fig. 2C). In addition, we also observed that the downregulation of Hh transcriptional activity and the expression of target genes induced by USP44 overexpression were impaired by Itch inhibition (Supplementary Fig. 2D–G), indicating that Itch is critical for USP44-mediated regulation of the Hh signaling pathway. Moreover, since the C-terminal PPXYs and pSP degron within the Gli1 protein are essential for the association of Itch [22], we assessed whether the regulatory effect of USP44 depended on the presence of this motif. Thus, wild-type Gli1 (His-Gli1 WT) or mutant Gli1 (His-Gli1 ∆C) lacking the motif needed for Itch interaction was transfected into USP44-knockdown HCC cells. As a result, we found that USP44 knockdown could led to an increase in the expression of wild-type (WT) Gli1 but not mutant Gli1 (Fig. 3Q). Taken together, these results strongly support that USP44 specifically modulates Itch-dependent Gli1 protein levels and thus inactivates the Hh signaling pathway and PDL1 expression.

### USP44 stabilizes Itch by deubiquitinating Itch

**Fig. 4:**
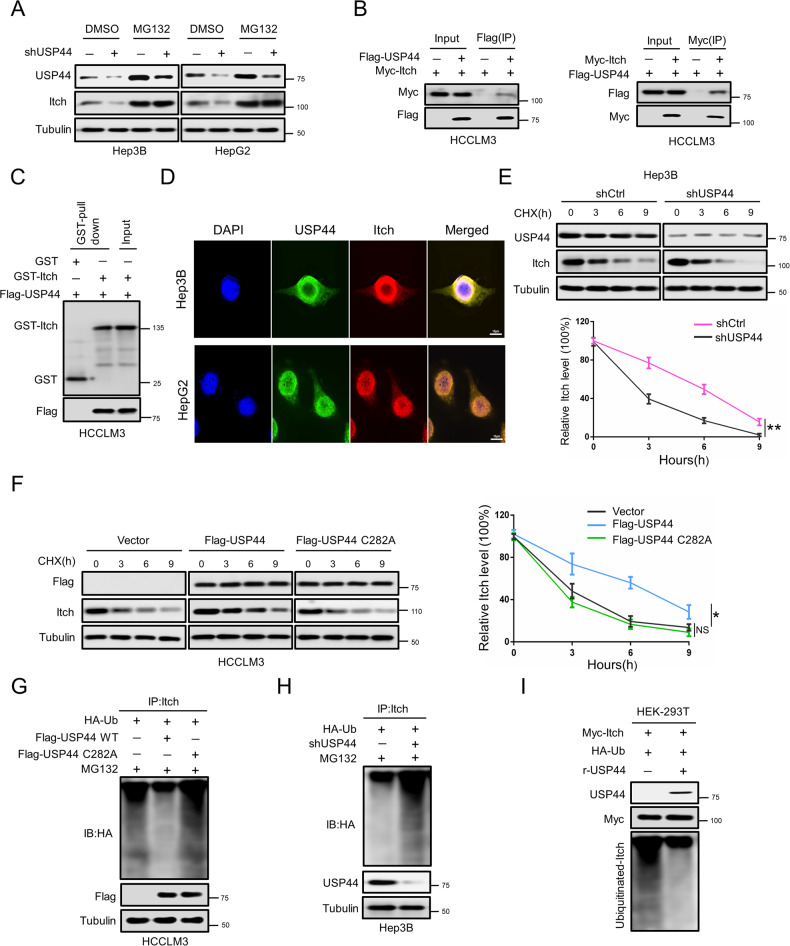
USP44 interacts with and deubiquitinates Itch. **A** Western blot for protein expression of Itch in shCtrl or shUSP44 transfected HCC cells treated with or without MG132(15 μM, 6 h). **B** HCCLM3 cells were transfected with Flag-USP44 and Myc-Itch. Interaction between USP44 and Itch was detected by immunoprecipitation followed by western blot analysis with the indicated antibodies. **C** GST pull-down assays showed that GST-labeled Itch could directly interact with Flag-labeled USP44. **D** Co-localization of USP44 and Itch detected by immunofluorescence. Green and red, USP44 and Itch expressing cells, respectively. Nuclei were stained blue with DAPI. Scale bar indicates 10 μm. **E** shCtrl or shUSP44 transfected Hep3B cells were treated with CHX (20 μg/ml). The samples were collected at indicated time points and subjected to western blot analysis. The amounts of lysates from shCtrl and shUSP44 transfected Hep3B cells were adjusted to achieve similar levels of Itch at 0 h. **F** Control vector or exogenous USP44 transfected HCCLM3 cells were treated with CHX (20 μg/ml). The samples were collected at indicated time points and subjected to western blot analysis. The amounts of lysates from control vector and exogenous USP44 transfected HCCLM3 cells were adjusted to achieve similar levels of Itch at 0 h. **G** HA-Ub and wild-type or mutant USP44 were co-transfected into HCCLM3 cells, which were treated with 15 μM MG132 for 6 h before being harvested. The cells were immunoprecipitated with an anti-Itch antibody, followed by western blot analysis. **H** HA-Ub and shCtrl or shUSP44 were co-transfected into Hep3B cells, which were treated with 15 μM MG132 for 6 h before being harvested. The cells were immunoprecipitated with an anti-Itch antibody, followed by western blot analysis. **I** Deubiquitination of Itch in vitro by recombinant USP44. HEK-293T cells transfected with Myc-Itch and HA-Ub were treated with MG132 (15 μM) for 6 h before harvest. Cell lysates were incubated with FLAG Sepharose, then ubiquitinated Itch was incubated with or without purified recombinant USP44. The ubiquitin on Itch was analyzed by Western blot. Data are obtained from three independent biological replicates and are presented as mean ± SD. *p < 0.05, **p < 0.01, ***p < 0.001 as indicated.

We then investigated how USP44 regulates the expression of Itch in HCC cells. qRT-PCR was performed to test the mRNA levels of Itch in USP44-downregulated or USP44-upregulated HCC cells. As shown in Supplementary Fig. 3A–D, neither USP44 downregulation nor upregulation affected the mRNA level of Itch. However, the results of western blot showed that the inhibition of Itch expression caused by USP44 knockdown was fully restored by treatment with the proteasome inhibitor MG132 (Fig. 4A), suggesting that USP44 controls the turnover of Itch via the proteasomal pathway.

Given that USP44 is a DUB that promotes the stability of substrates, we sought to investigate whether USP44 promoted Itch stabilization through a deubiquitination event. Thus, we first co-transfected exogenous USP44 and Itch into HCC cells, and the reciprocal co-immunoprecipitation (co-IP) assay showed that USP44 and Itch interacted with each other (Fig. 4B). The direct association between USP44 and Itch was further verified by a GST pull-down experiment (Fig. 4C). Moreover, we employed immunofluorescence assays, and the immunofluorescence signals showed the endogenous USP44 and Itch proteins in HCC cells. Our results revealed that USP44 was colocalized with Itch (Fig. 4D), suggesting that USP44 interacts with Itch in HCC cells. Next, we investigated whether USP44 regulates the stabilization of Itch. Following treatment with cycloheximide (CHX), Itch displayed a shorter half-life in the USP44-downregulated group than in the control group (Fig. 4E, Supplementary Fig. 3E). Consistently, the overexpression of WT USP44, but not the deubiquitinase inactive mutant of USP44(C282A), was sufficient to induce an extended half-life of the Itch protein (Fig. 4F, Supplementary Fig. 3F), indicating that USP44 stabilized the expression of Itch in a deubiquitinase activity-dependent manner. These findings prompted us to evaluate the ubiquitination level of Itch controlled by USP44. We observed that high levels of USP44, but not USP44(C282A), significantly inhibited the ubiquitination level of Itch (Fig. 4G). Conversely, USP44-knockdown robustly increased the level of ubiquitinated Itch (Fig. 4H). Furthermore, we conducted an in vitro deubiquitination assay by purifying ubiquitinated Itch from cells expressing Myc-Itch and HA-Ub and incubating recombinant USP44 with ubiquitinated Itch in a cell-free system. The addition of recombinant USP44 effectively deubiquitinated Itch in vitro (Fig. 4I). Overall, these data validated that USP44 deubiquitinates and stabilizes Itch by functioning as a DUB.

### USP44 inhibits the proliferation and migration of HCC cells by suppressing the Hh signaling pathway in vitro

**Fig. 5:**
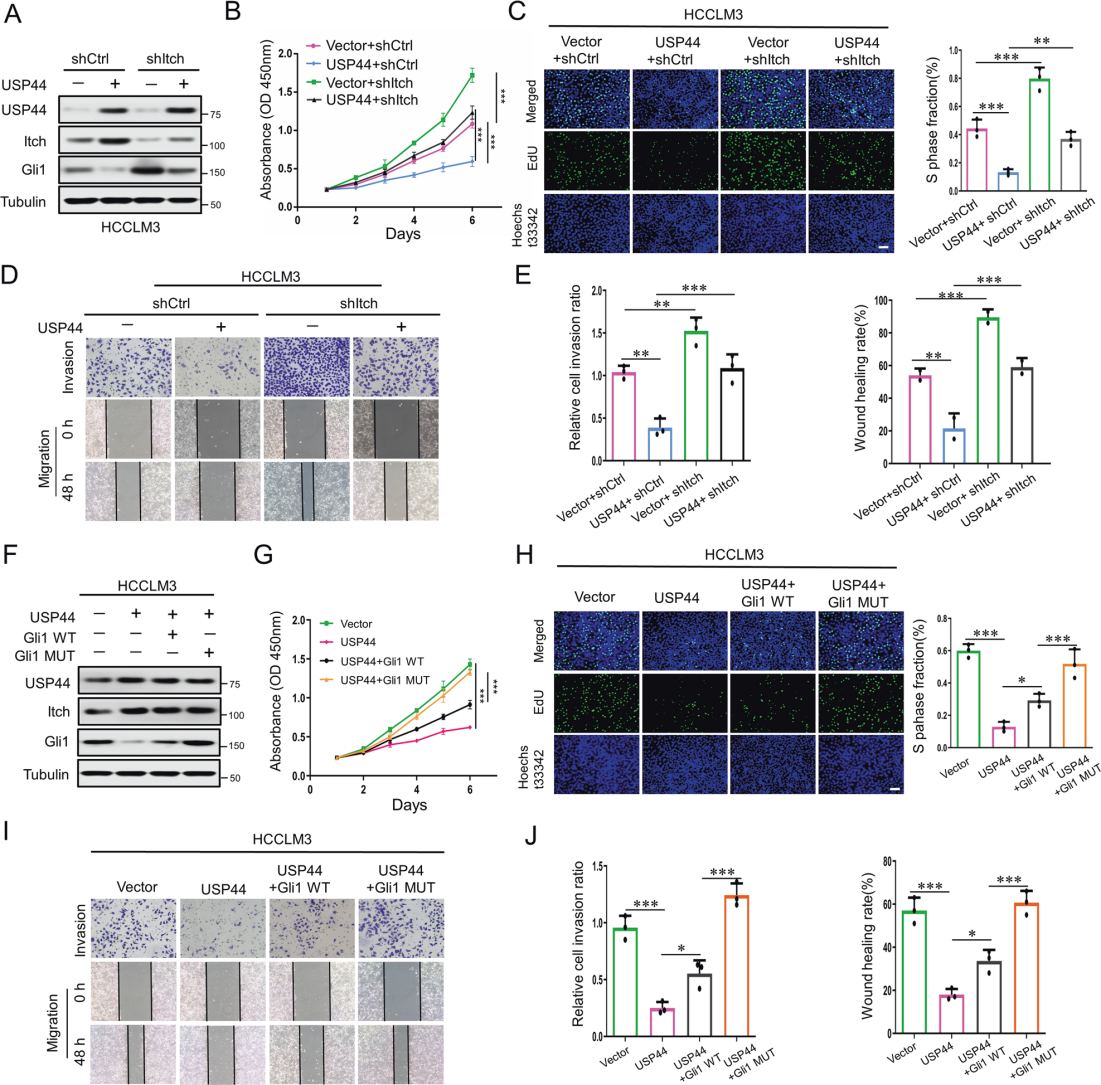
USP44 impairs Hh signaling-dependent proliferation and migration of HCC cells in vitro. **A** Itch and Gli1 protein expression in HCCLM3 cells expressing control vector or exogenous USP44, with or without Itch knockdown. **B**, **C** CCK-8 and EdU analyses for cell proliferation in HCCLM3 cells expressing control vector or exogenous USP44, with or without shItch transfection. Scale bar: 100 μm. **D**, **E** Transwell and wound-healing assays were used to test cellular invasion and migration in HCCLM3 cells expressing control vector or exogenous USP44, with or without shItch transfection. **F** Itch and Gli1 protein expression in control vector or exogenous USP44 expressing HCCLM3 cells transfected with wild-type Gli1 or the mutant Gli1. **G,** **H** CCK-8 and EdU analyses for cell proliferation in control vector or exogenous USP44 expressing HCCLM3 cells transfected with wild-type Gli1 or the mutant Gli1. Scale bar: 100 μm. **I**, **J** Transwell and wound-healing assays were used to test cellular invasion and migration in control vector or exogenous USP44 expressing HCCLM3 cells transfected with wild-type Gli1 or the mutant Gli1. Data are obtained from three independent biological replicates and are presented as mean ± SD. *p < 0.05, **p < 0.01, ***p < 0.001 as indicated.

Because our findings demonstrate that USP44 is critical for Hh signaling activity and that Hh signaling is known to play important roles in the modulation of tumors’ malignant behavior, we examined whether USP44 could influence HCC progression by regulating Hh signaling. Therefore, we first evaluated the effect of USP44 on the proliferative ability of HCC cells. The results from CCK-8 and EdU assays showed that the upregulation of USP44 significantly inhibited cell growth, while Itch knockdown impaired the decline in cell proliferation induced by USP44 overexpression (Fig. 5A–C). In contrast, the knockdown of USP44 enhanced cell growth, whereas Itch overexpression mitigated the increase in cell proliferation enhanced by USP44 knockdown (Supplementary Fig. 4A–C). In addition, wound healing and transwell assays were used to measure the effects of USP44 on the invasion and migration of HCC cells. As a result, USP44 overexpression suppressed cellular invasion and migration, while downregulation of Itch alleviated the inhibition of cellular invasion and migration induced by USP44 overexpression (Fig. 5D, E). USP44-knockdown promoted cellular invasion and migration, whereas the overexpression of Itch diminished the increase in cellular invasion and migration caused by USP44 knockdown (Supplementary Fig. 4D, E). These data indicated that USP44 inhibited proliferation, invasion, and migration by modulating the expression of Itch in HCC cells.

Furthermore, to validate that the Hh signaling pathway participates in the regulation of USP44 on the malignant behavior of HCC cells, we introduced WT Gli1 and mutant Gli1 into USP44-overexpressing HCC cells. Mutant Gli1 was constructed by generating point mutations of Ser102(S102), Ser102(S408), Ser481(S481) and Thr107(T1074) to alanine. Mutation of these four sites into alanine prevents its phosphorylation and nuclear import, which consequently facilitates the stabilization of Gli1 and activation of Hh signaling. As shown in Fig. 5F, mutant Gli1, but not WT Gli1, escaped Itch-mediated degradation promoted by USP44 overexpression. Moreover, the introduction of mutant Gli1 significantly reversed the reduction in cellular proliferation, invasion, and migration caused by USP44 overexpression (Fig. 5G–J). Together, these findings suggested that overexpression of USP44 inhibited cellular proliferation and migration through inhibition of the Hh pathway in vitro.

### USP44 impairs the growth and metastasis of HCC by inhibiting the Hh signaling pathway in vivo

**Fig. 6:**
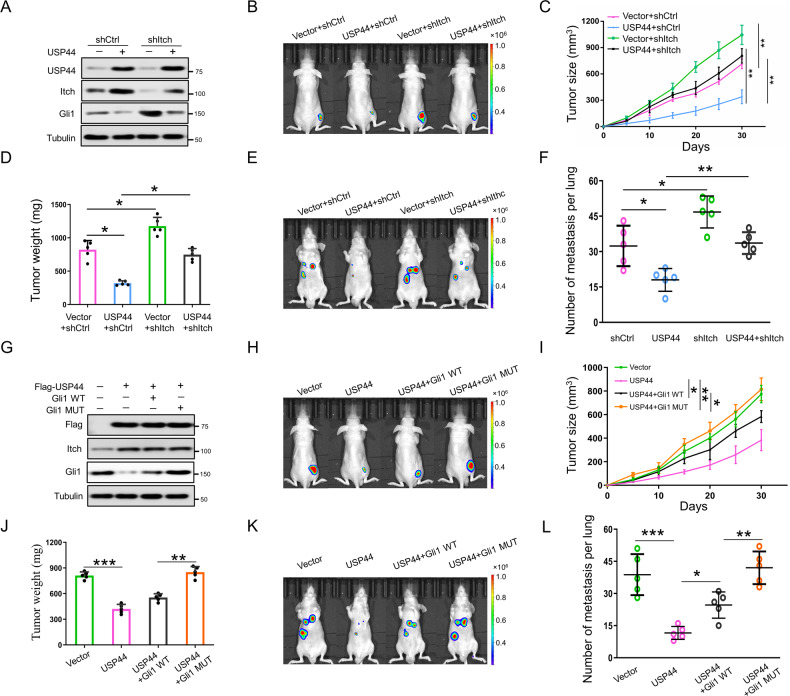
USP44 inhibits Hh signaling-dependent growth and metastasis of HCC in vivo. **A** Nude mice were subcutaneously injected with HCCLM3 cells stably expressing indicated plasmids. Intratumor protein levels of Itch and Gli1 in indicated groups were detected by western blot. **B** BALB/C nude mice subcutaneously injected with luciferase-expressing HCCLM3/Vector+shCtrl, HCCLM3/USP44+shCtrl, HCCLM3/Vector+shItch, or HCCLM3/USP44+shItch cells were assessed by IVIS imaging system. **C**, **D** Volume and weight of tumors in indicated groups (*n* = 5 per group). **E** IVIS imaging system was used to detect the effect of USP44 on metastasis of HCC in nude mice with orthotopic implanted xenografts. **F** Quantification of lung metastasis in the indicated groups of mice (*n* = 5 per group). **G** Nude mice were subcutaneously injected with HCCLM3 cells stably expressing indicated plasmids. Intratumor protein levels of Itch and Gli1 in indicated groups were detected by western blot. **H** Nude mice subcutaneously injected with luciferase-expressing HCCLM3/Vector, HCCLM3/USP44, HCCLM3/USP44+Gli1 WT, or HCCLM3/USP44+Gli1 MUT cells were assessed by IVIS imaging system. WT, wild-type; MUT, mutant. **I**, **J** Volume and weight of tumors in HCCLM3/Vector, HCCLM3/USP44, HCCLM3/USP44+Gli1 WT, or HCCLM3/USP44+Gli1 MUT groups. **K** IVIS imaging system was used to detect the effect of USP44 on metastasis of HCC in nude mice with orthotopic implanted xenografts. **L** Quantification of lung metastasis in the indicated groups of mice (*n* = 5 per group). Data are obtained from three independent biological replicates and are presented as mean ± SD. *p < 0.05, **p < 0.01, ***p < 0.001 as indicated.

To evaluate the role of USP44 during HCC progression in vivo, we subcutaneously implanted HCCLM3 cells stably expressing USP44 vectors into nude mice. Consistent with the results observed in cell culture experiments, we found that the intratumor protein levels of Itch were evidently increased while Gli1 expression was dramatically decreased in the USP44-overexpressing group compared with the control group. Knockdown of Itch impaired the decline in the intratumor expression of Gli1 induced by USP44 overexpression (Fig. 6A). In addition, USP44 overexpression inhibited the growth of the tumors whereas the downregulation of Itch blunted the decline in tumor growth (Fig. 6B–D). To determine the effect of USP44 on HCC metastasis, orthotopic implanted xenografts in nude mice were employed. USP44 overexpression reduced the metastatic potential, whereas Itch knockdown reversed the decline in the metastatic potential caused by USP44 overexpression (Fig. 6E, F). These data collectively suggested that Itch upregulation is important in USP44 overexpression-mediated tumor growth and metastasis inhibition in vivo.

Next, we sought to verify that the Hh signaling pathway was involved in the modulatory effect of USP44 on tumor growth and metastasis in vivo. We examined the effect of a nuclear-residing Gli1 mutant on USP44 overexpression-mediated tumor inhibition. Consistent with the aforementioned findings, the intratumor protein levels of WT Gli1, but not the mutant Gli1, were inhibited by USP44 overexpression (Fig. 6G). In addition, the upregulation of USP44 delayed tumor growth and lung metastasis while the introduction of mutant Gli1 attenuated the suppression of tumor growth and metastasis mediated by USP44 overexpression (Fig. 6H–L). Collectively, these data suggest that the regulatory effects on the Hh pathway are critical for USP44 to arrest the growth and metastasis of HCC in vivo.

### GANT61 improves the anti-PDL1 therapeutic efficacy by targeting Gli1

**Fig. 7:**
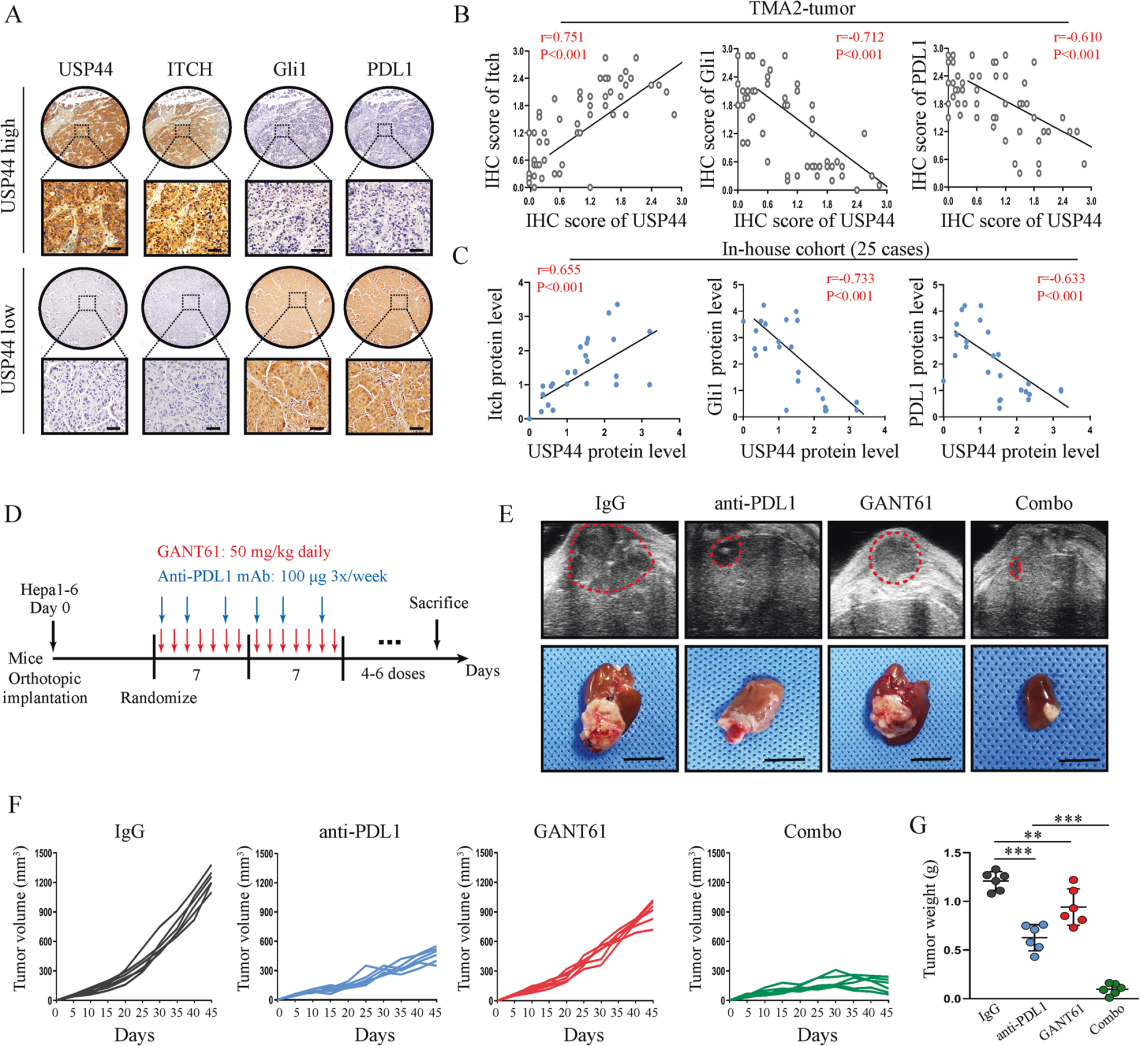
GANT61 promotes the PDL1 therapy efficacy by targeting Gli1. **A**, **B** Representative images and IHC score of IHC staining for indicated proteins in USP44-high and USP44-low HCC samples. Scale bar: 50 μm. **C** Correlations between USP44 and indicated protein levels detected from HCC tissues of 25 patients. **D** Schematic illustration of the therapy schedule for GANT61, anti-PDL1 mAb or combination therapy in orthotopic HCC mouse model using Hepa1-6 cell line. **E**–**G** Representative images are presented at the endpoint and periodical detection of tumor development and progression using an ultrasound imaging platform (*n* = 6 per group). *p < 0.05, **p < 0.01, ***p < 0.001 as indicated.

To investigate the clinical significance of the USP44-Itch-Gli1-PDL1 axis and identify their correlation in HCC, we tested their expression in HCC tissue specimens by performing IHC staining and western blot. In TMA2, high levels of USP44, and Itch and low expression of Gli1 and PDL1 occurred frequently and concomitantly (Fig. 7A, B). We also examined the protein levels of USP44, Itch, Gli1 and PDL1 in 25 of our in-house HCC tissues and found consistent results (Fig. 7C).

We further introduced GANT61, an inhibitor of Gli1 [25, 26]. By establishing an orthotopic syngeneic model of HCC using Hepa1-6 cells, we systemically administered GANT61 and an anti-PDL1 monoclonal antibody (mAb) (Fig. 7D). Our results indeed confirmed the efficacy of the combination therapy of GANT61 and anti-PDL1 mAb. GANT61 and anti-PDL1 mAb combination therapy effectively inhibited tumor growth and prolonged the survival rate of mice compared with the control and monotherapy groups (Fig. 7E–G, Supplementary Fig. 5A). In addition, there were no significant changes in serum indicators of liver and kidney function among the different groups of mice during treatment, indicating that this approach may have limited damage to the liver and kidney function of mice (Supplementary Fig. 5B, C). Collectively, our findings demonstrated that targeting Gli1 effectively enhanced immune checkpoint blockade in preclinical models of HCC.

## Discussion

HCC is a malignant tumor with poor prognosis, and exploring regulatory targets is a crucial challenge in the treatment of HCC. Increasing evidence has shown that aberrant activation of Hh signaling is closely associated with a multitude of solid tumor types, including HCC [27, 28]. Hh signaling is considered to regulate the process of hepatic carcinogenesis, progression, metastasis and drug resistance through the activation of the downstream effector protein Gli1 [29, 30]. Interestingly, emerging evidence has shown that Gli1 also functions as a positive regulator of PDL1 expression, highlighting the potential importance of Hh signaling in tumor immunotherapy [20]. Therefore, probing upstream regulators of Hh signaling has emerged as an attractive option to develop potential therapeutic strategies. Herein, we revealed that USP44 may exhibit a tumor suppressive role in HCC progression through inhibiting the Hh pathway.

USP44 is a member of the ubiquitin-specific protease family that is able to remove ubiquitin modifications from its substrates and thus protects substrates from proteolysis. Previous reports have demonstrated that USP44 plays diverse roles in the initiation and development of cancer [31–34]. A recent study suggested that low USP44 expression is associated with poorer survival and a later tumor stage in HCC [35]. However, the role of USP44 in HCC progression requires further investigation. In this study, we originally found that USP44 is downregulated in HCC tissues and that low USP44 expression is correlated with poor prognosis in patients with HCC. Subsequently, both in vitro and in vivo experiments demonstrated that USP44 inhibits the proliferative and invasive capacity of HCC by inactivating Hh signaling.

Gli1 is a terminal effector of the Hh cascade and elicits signal amplification by acting as a transcription factor [30]. The expression of Gli1 is well regulated by several types of posttranslational modifications, including ubiquitination [36, 37]. Itch is a well-characterized E3 ligase that binds with Gli1 at its C-terminus, thereby facilitating its polyubiquitylation and proteasomal degradation. Indeed, increasing evidence has revealed a tumor suppressor role of Itch through antagonizing the activation of the Gli1-dependent Hh signaling pathway [32]. Nevertheless, Itch is frequently maintained in an inactive state in tumor due to its autoubiquitylation through K48 linkage [32, 33]. Hence, it is important to explore DUBs that stabilize the expression of Itch, thus limiting the oncogenic properties of Gli1 and then maintaining Hh signaling off. In this work, we demonstrated that USP44 inactivates Hh signaling by deubiquitinating and stabilizing Itch, which in turn induces the ubiquitylation and proteolysis of Gli1. This conclusion was based on the observations discussed below. First, wild-type USP44, but not the deubiquitinase inactive mutant, was identified to interact with Itch and remove the polyubiquitin chains from Itch. Second, USP44 promoted the stabilization of Itch and in turn led to Gli1 degradation. However, when the Gli1 motif (PPXYs and pSP degron) which is essential for the association of Itch was mutated, the decrease in Itch induced by USP44 downregulation failed to stabilize Gli1 expression. In addition, as both Itch downregulation and Gli1 overexpression were able to reverse the carcinostatic role of USP44, we suggested that USP44 was able to inhibit HCC growth and metastasis through inactivating Hh signaling in an Itch-dependent manner.

Notably, several studies have reported that the cross-talk between DUB and E3 ligases plays a critical role in regulating cellular protein dynamics [38–41]. The interaction of DUB and E3 ligases has the potential to facilitate multifaceted results, including E3 ligase antagonization, E3 ligase deubiquitylation, and polyubiquitin chain editing on substrates [41]. For example, several DUBs, including USP2, USP12, and USP26 have been reported to promote the expression of the E3 ubiquitin ligase MDM2 and consequently result in the degradation of the substrate protein p53 [38–40]. Another striking example involves the WNT signal transducing protein disheveled (DVL2), which is coordinately controlled by the E3 ubiquitin ligase WWP1 and deubiquitylase USP9X. The coupling of DUB and E3 activities on DVL2 establishes a ubiquitin rheostat that determines its participation in either canonical WNT or noncanonical WNT signaling [41]. Here, we identified that USP44 interacts with Itch to form a DUB-E3 complex and stabilize the expression of Itch, thereby hampering Gli1-mediated Hh signaling activation and PDL1 expression. Clinically, we observed that USP44 possessed a positive correlation with Itch expression but a negative correlation with Gli1 or PDL1 expression in HCC. In addition, the Gli1 inhibitor GANT61, which has previously been shown to inhibit the progression of several tumors [25, 26], has been shown to significantly improve anti-PDL1 efficacy in a murine model. Nevertheless, future studies will be necessary to develop USP44 activators to confirm the anticarcinogenic role of USP44 in the progression of HCC.

In summary, in this study we identified USP44 as a novel deubiquitinating modulator that inhibits Hh signaling and PDL1 expression in HCC. USP44 deubiquitinates and stabilizes Itch and thus promotes Itch-mediated degradation of Gli1. The downregulated expression of USP44 in HCC tissues drives sustained activation of Hh signaling and the downstream oncogenic response. Therefore, our study provides a new perspective for HCC intervention by targeting the USP44-Itch-Gli1 signaling axis.

### Supplementary information

Original Data File

Checklist

Supplementary Figures

Supplementary Materials and Methods

Table S1

Table S2

## Data Availability

All data relevant to the study are included in the paper or uploaded as supplementary material. The data and sources associated with this study are available from the corresponding author upon reasonable request.
