# Supplementary material for: The deubiquitinating enzyme USP44 suppresses hepatocellular carcinoma progression by inhibiting Hedgehog signaling and PDL1 expression
Source: Cell Death Dis. 2023 Dec 14;14(12):830. doi: 10.1038/s41419-023-06358-y (PMC10721641; doi:10.1038/s41419-023-06358-y)

Figure 1B

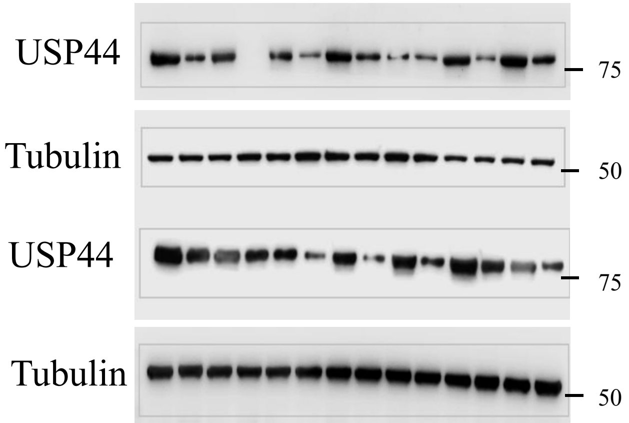

Figure 2E

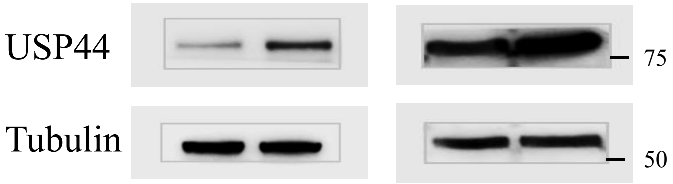

Figure 3I

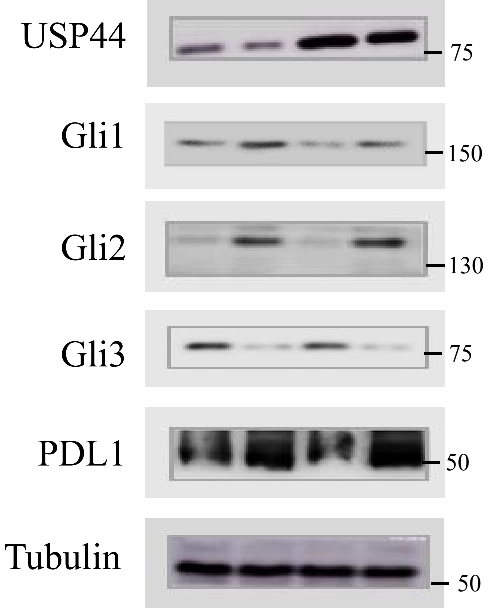

Figure 3M

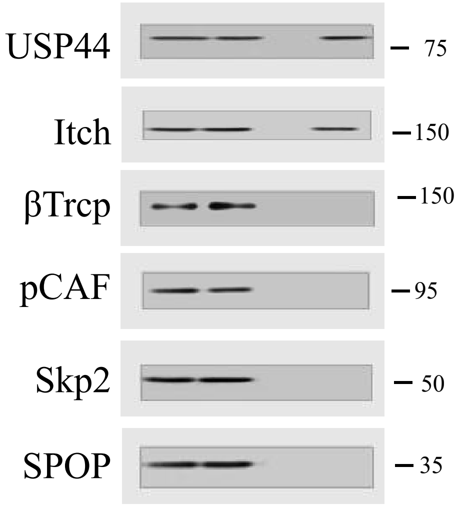

Figure 3E

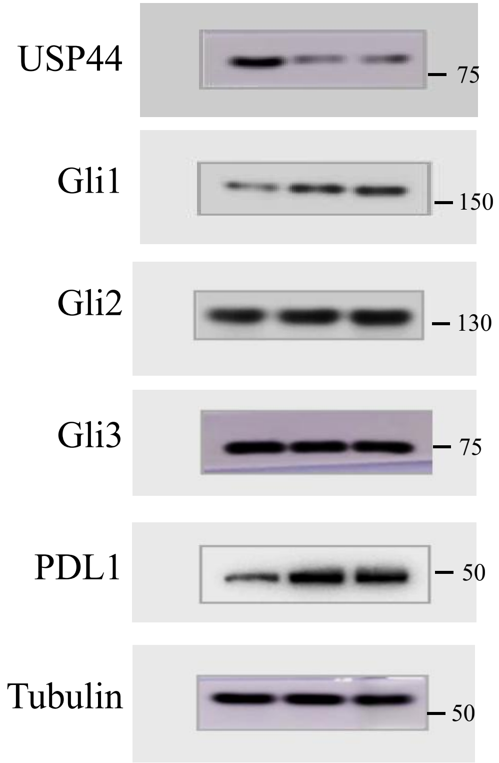

Figure 2A

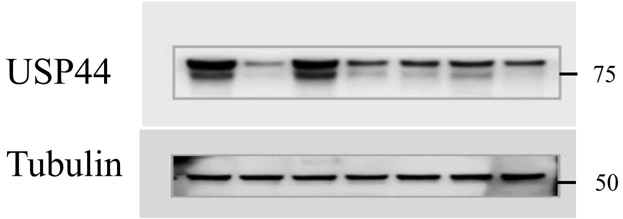

Figure 3L

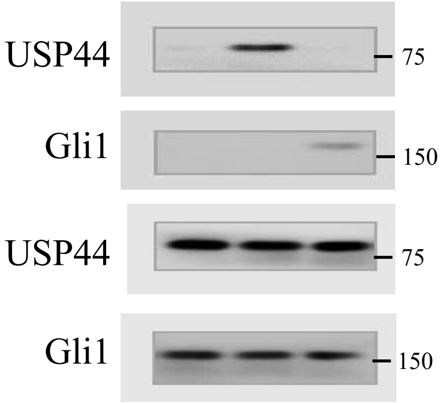

Figure 2C

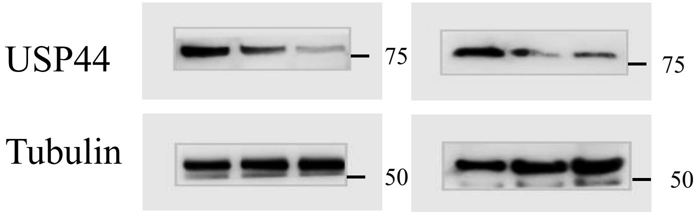

Figure 3N

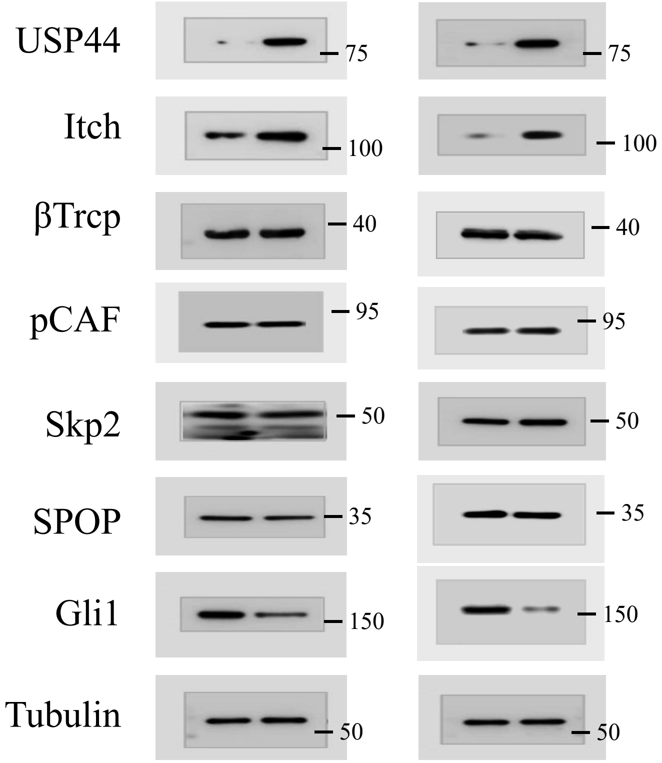

Figure 3O

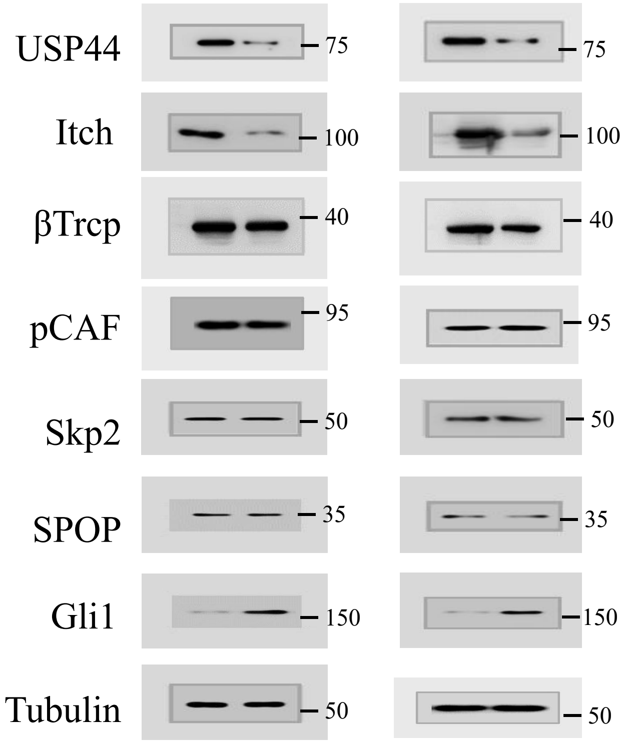

Figure 4A

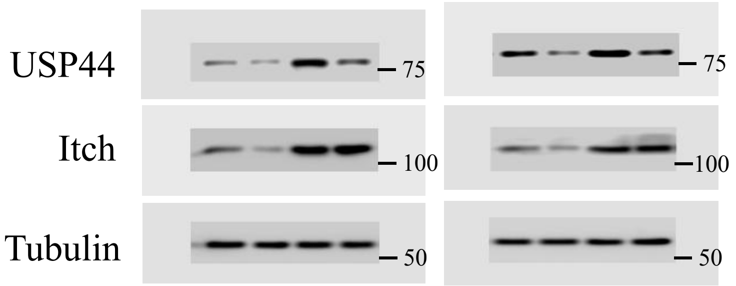

Figure 4B

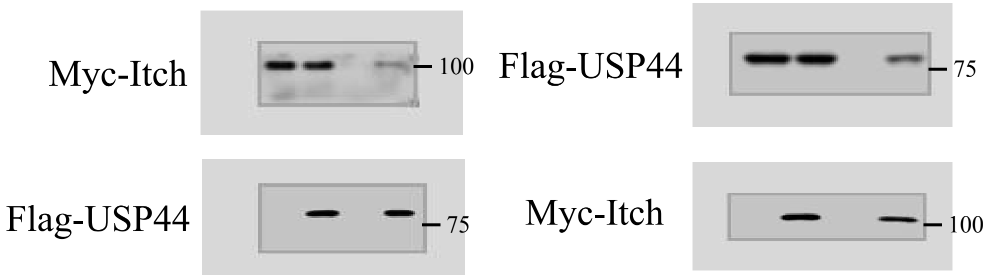

Figure 4C

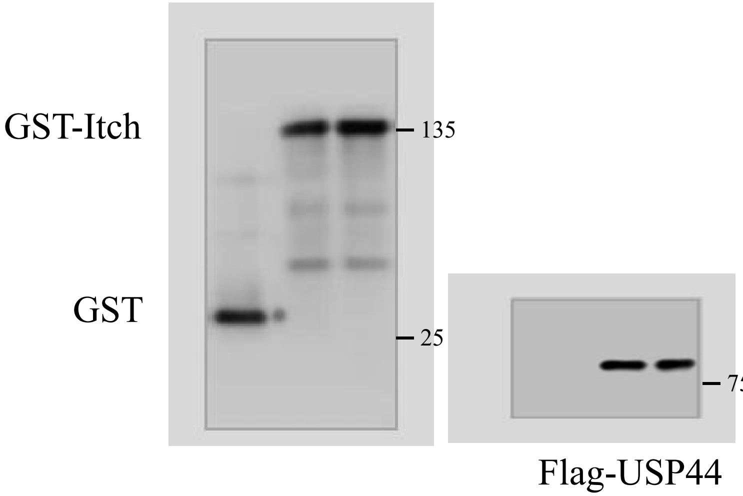

Figure 3P

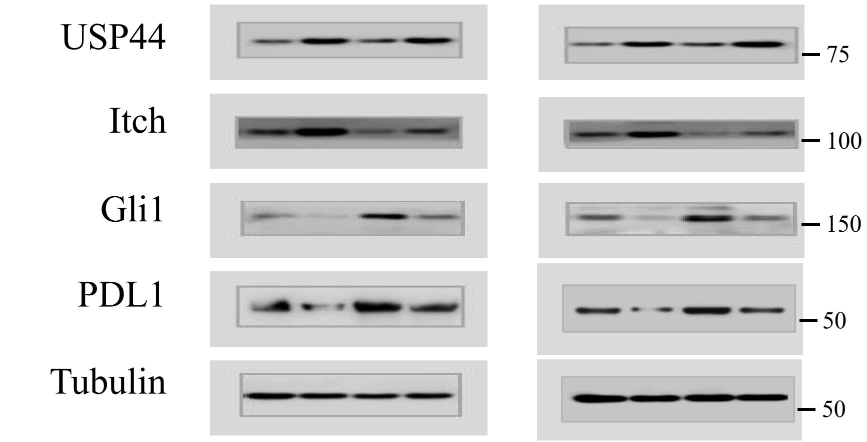

Figure 3Q

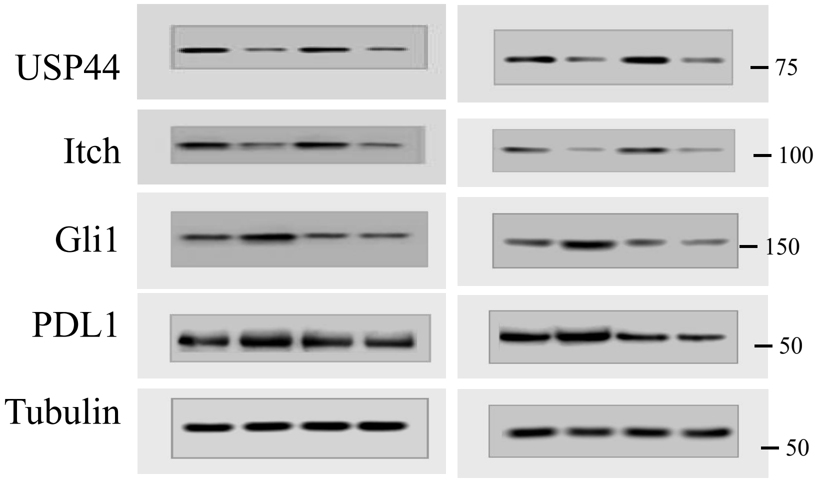

Figure 4E

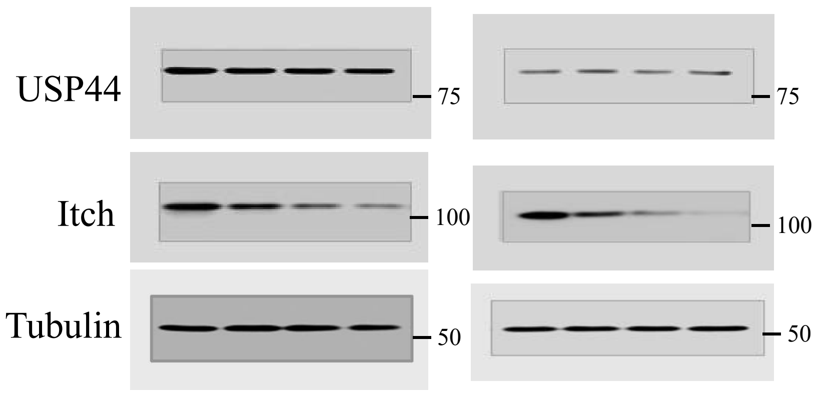

Figure 4G

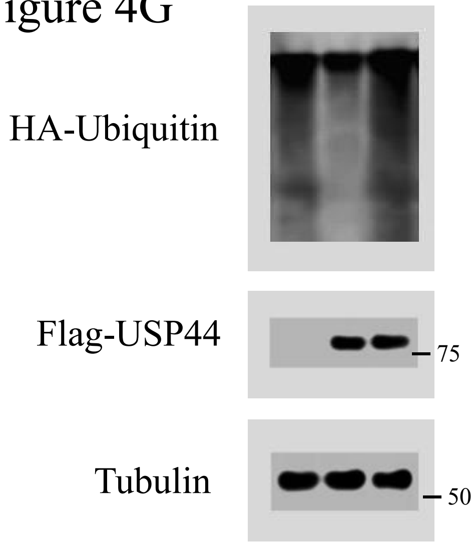

Figure 4H

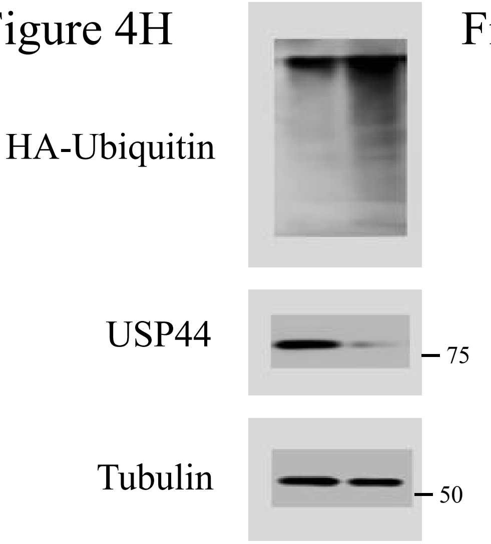

Figure 4I

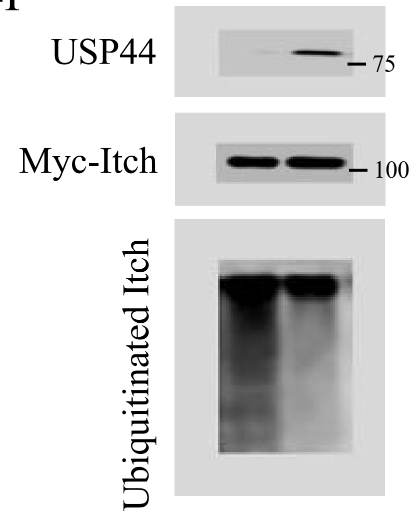

Figure 4F

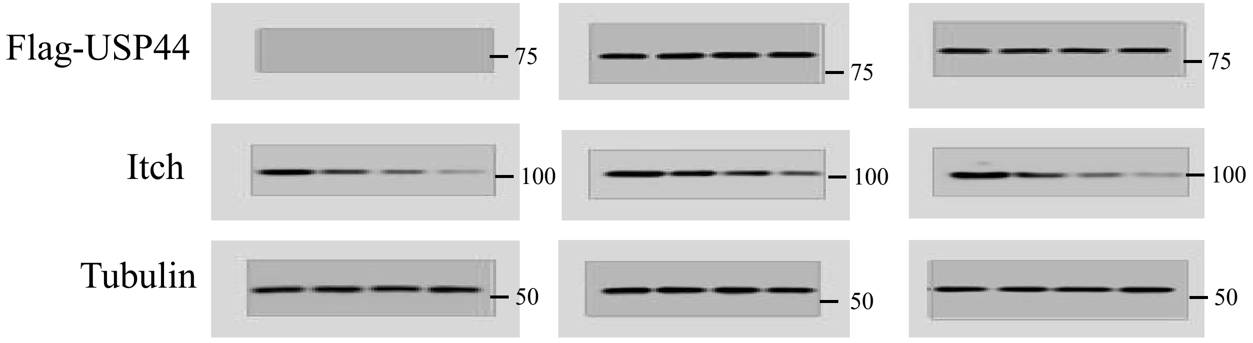

Figure 5A

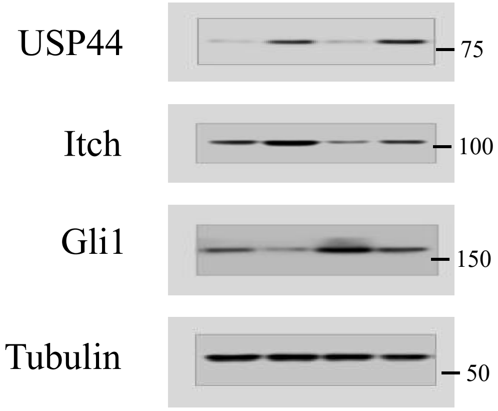

Figure 5F

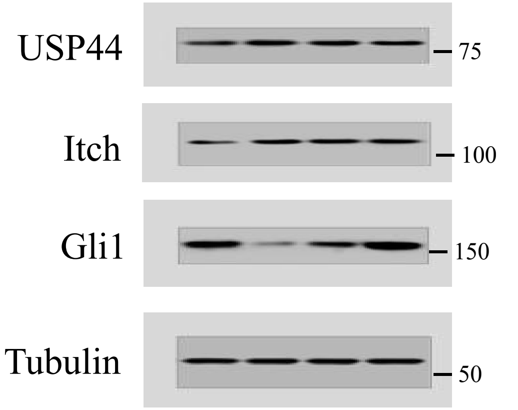

Figure 6A

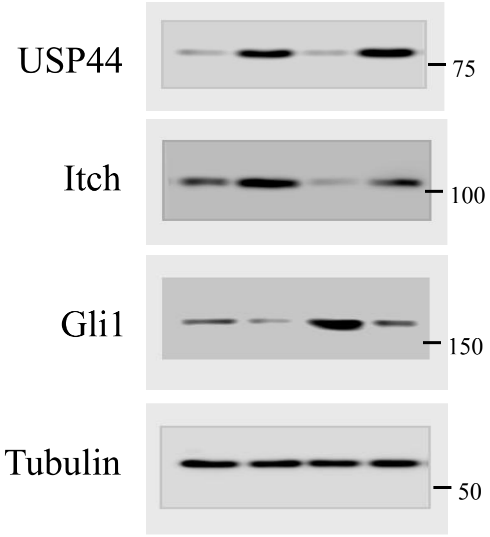

Figure 6G

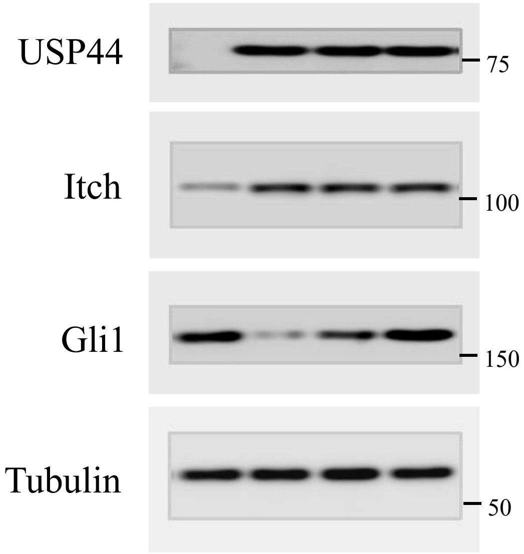

Supplement: Supplementary file 1 — Original Data File [file 41419_2023_6358_MOESM1_ESM.pdf]
