# Supplementary material for: The deubiquitinating enzyme USP44 suppresses hepatocellular carcinoma progression by inhibiting Hedgehog signaling and PDL1 expression
Source: Cell Death Dis. 2023 Dec 14;14(12):830. doi: 10.1038/s41419-023-06358-y (PMC10721641; doi:10.1038/s41419-023-06358-y)
Supplement: Supplementary file 3 — Supplementary Figures [file 41419_2023_6358_MOESM3_ESM.docx]

**Supplementary figure**

**
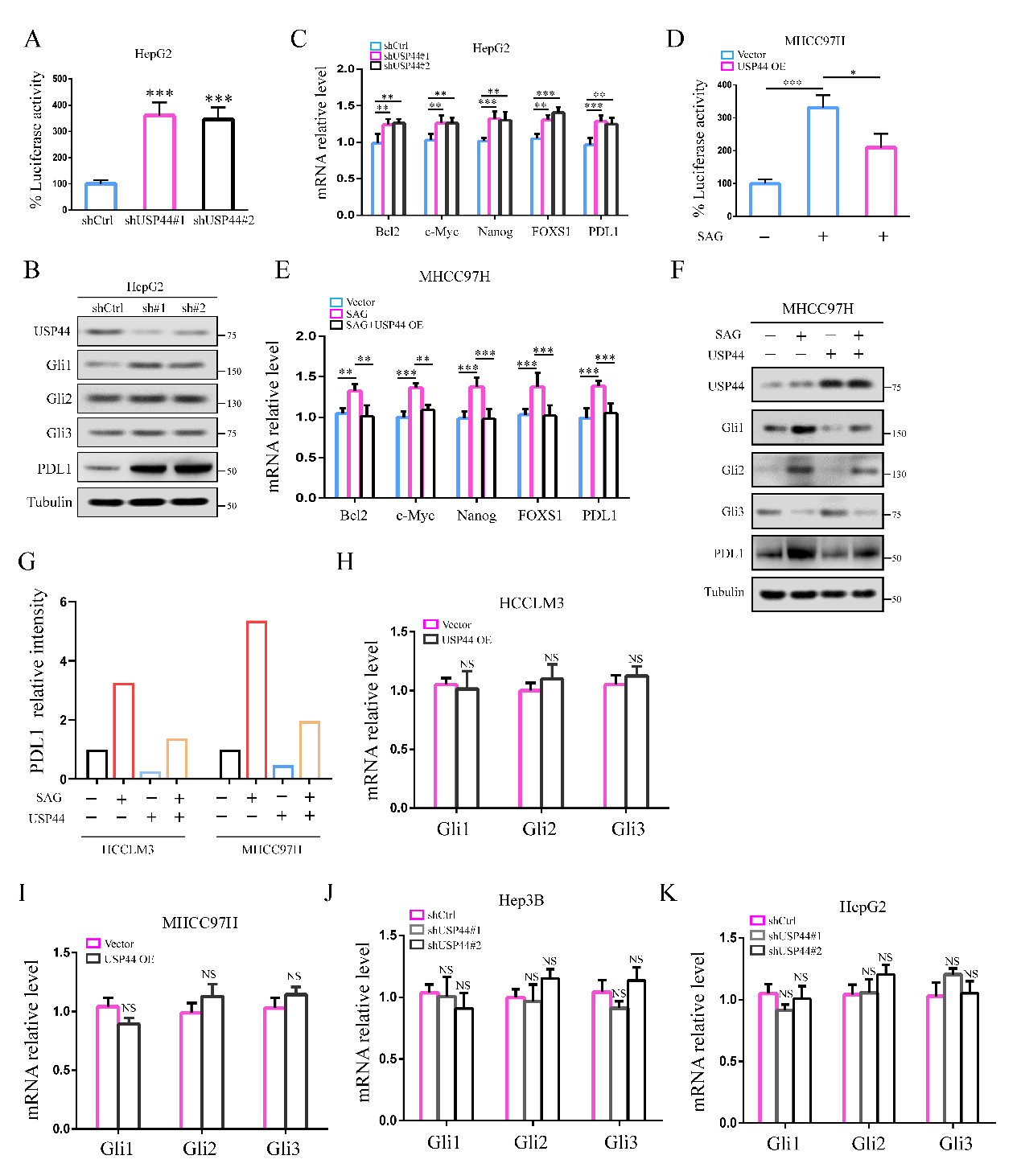
**

**Supplementary Fig. 1 USP44 negatively regulates the Hh pathway.**

**a** Luciferase activity of the Hh pathway-reporter in shCtrl or shUSP44 transfected HepG2 cells. **b** Western blot for protein expression of Gli1, Gli2, Gli3 and PDL1 in shCtrl or shUSP44 transfected HCC cells. **c** mRNA level of Hh signaling target genes in shCtrl or shUSP44 transfected HepG2 cells. **d** Luciferase activity of the Hh pathway-reporter in control vector or exogenous USP44 overexpressing MHCC97H cells treated with or without SAG (200 nM for 24 h). **e** mRNA level of Hh signaling target genes in control vector or exogenous USP44 overexpressing MHCC97H cells treated with or without SAG (200 nM for 24 h). **f** Western blot for protein expression of Gli1, Gli2, Gli3 and PDL1 in control vector or exogenous USP44 overexpressing MHCC97H cells treated with or without SAG (200 nM for 24 h). **g** Quantification of PDL1 expression in f and Figure 3i. **h,i** mRNA level of Gli1, Gli2 and Gli3 in HCC cells expressing control vector or exogenous USP44. **j, k** mRNA level of Gli1, Gli2 and Gli3 in shCtrl or shUSP44 transfected HCC cells. Data are obtained from three independent biological replicates and are presented as mean±SD. *p <0.05, **p <0.01, ***p <0.001 as indicated.


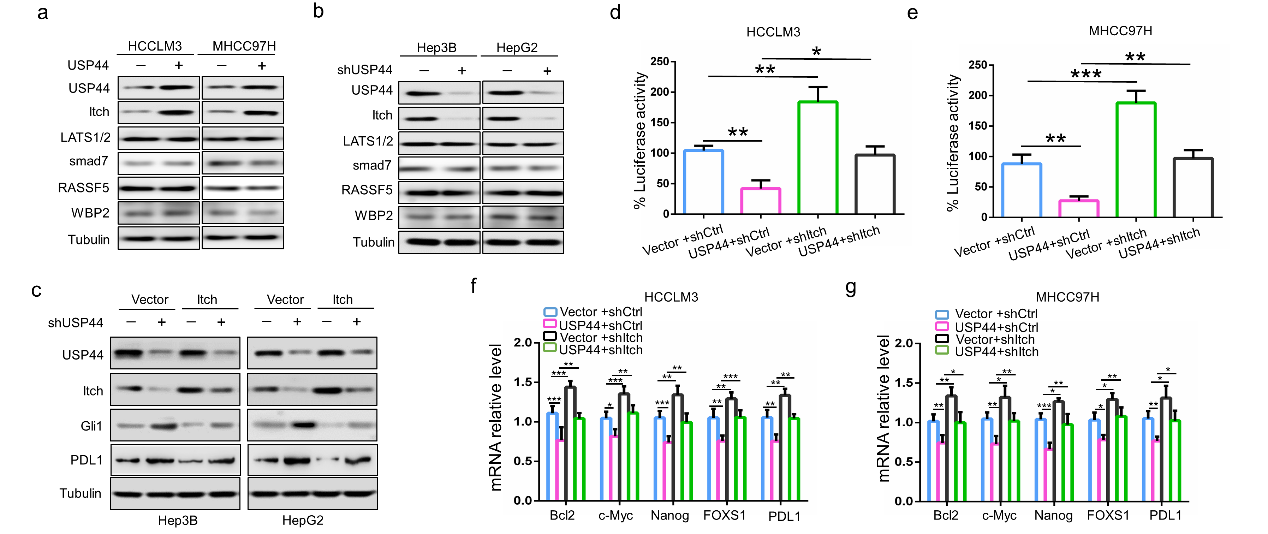


**Supplementary Fig. 2 USP44 inactivates Hh signaling through Itch.**

**a** Immunoblot for protein expression of Itch and its substrates in control vector or exogenous USP44 overexpressing HCC cells. **b** Immunoblot for protein expression of Itch and its substrates in shCtrl or shUSP44 transfected HCC cells. **c** Immunoblot for protein expression of Gli1 in USP44 knockdown HCC cells, with or without Itch overexpression. **d, e** Luciferase activity of the Hh pathway-reporter in HCC cells expressing control vector or exogenous USP44, with or without shItch transfection. **f, g** mRNA level of Hh target genes in HCC cells expressing control vector or exogenous USP44, with or without shItch transfection. Data are obtained from three independent biological replicates and are presented as mean±SD. *p <0.05, **p <0.01, ***p <0.001 as indicated.


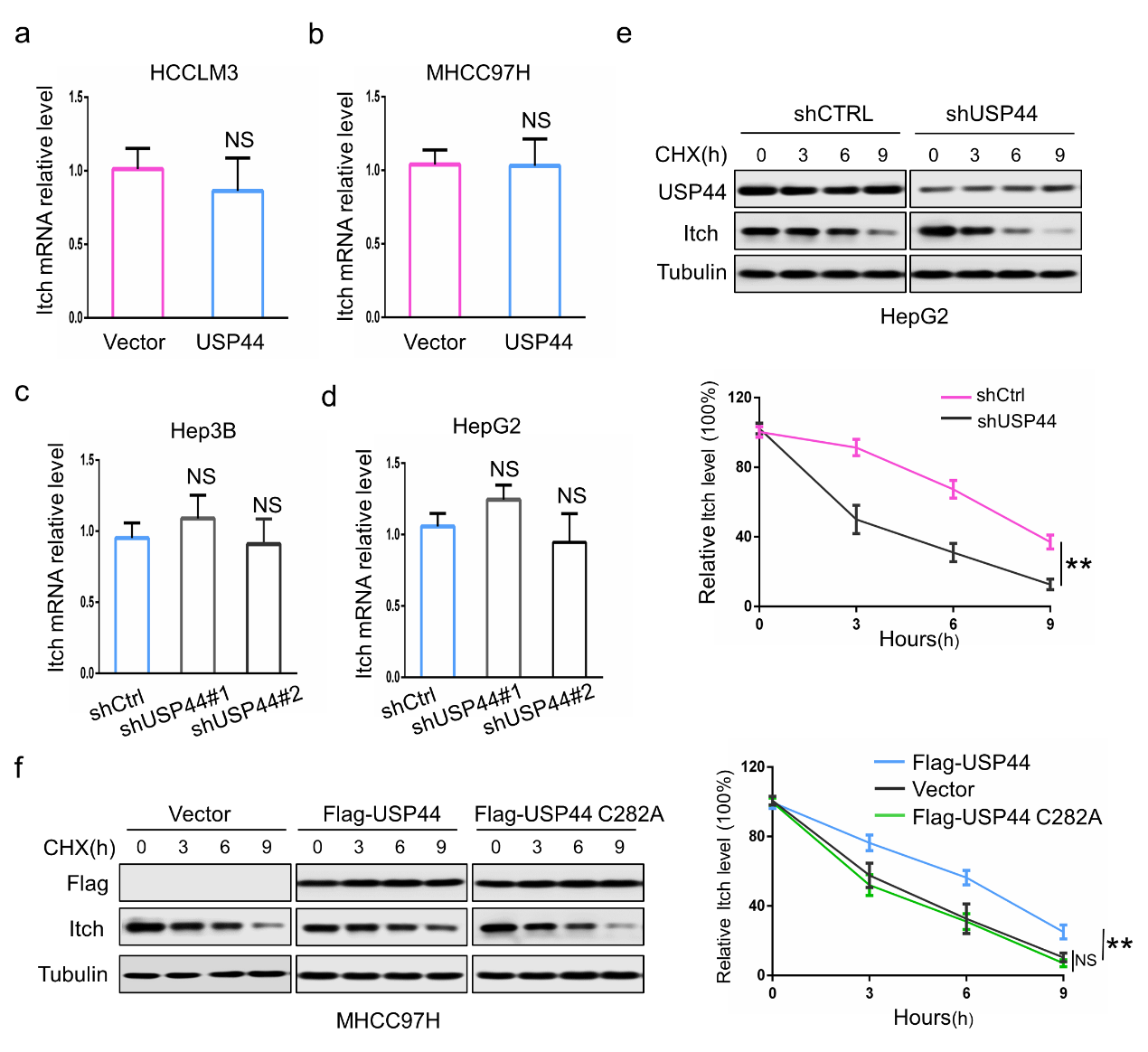


**Supplementary Fig. 3 USP44 upregulates and stabilizes the Itch protein**

**a, b** mRNA level of Itch in control vector or exogenous USP44 overexpressing HCC cells. **c, d** mRNA level of Itch in shCtrl or shUSP44 transfected HCC cells. **e** shCtrl or shUSP44 transfected HepG2 cells were treated with CHX (20 μg/ml). The samples were collected at indicated time points and subjected to western blot analysis. The amounts of lysates from shCtrl or shUSP44 transfected HepG2 cells were adjusted to achieve similar levels of Itch at 0 hour. **f** Control vector or exogenous USP44 transfected MHCC97H cells were treated with CHX (20 μg/ml). The samples were collected at indicated time points and subjected to western blot analysis. *p <0.05, **p <0.01, ***p <0.001 as indicated.

**
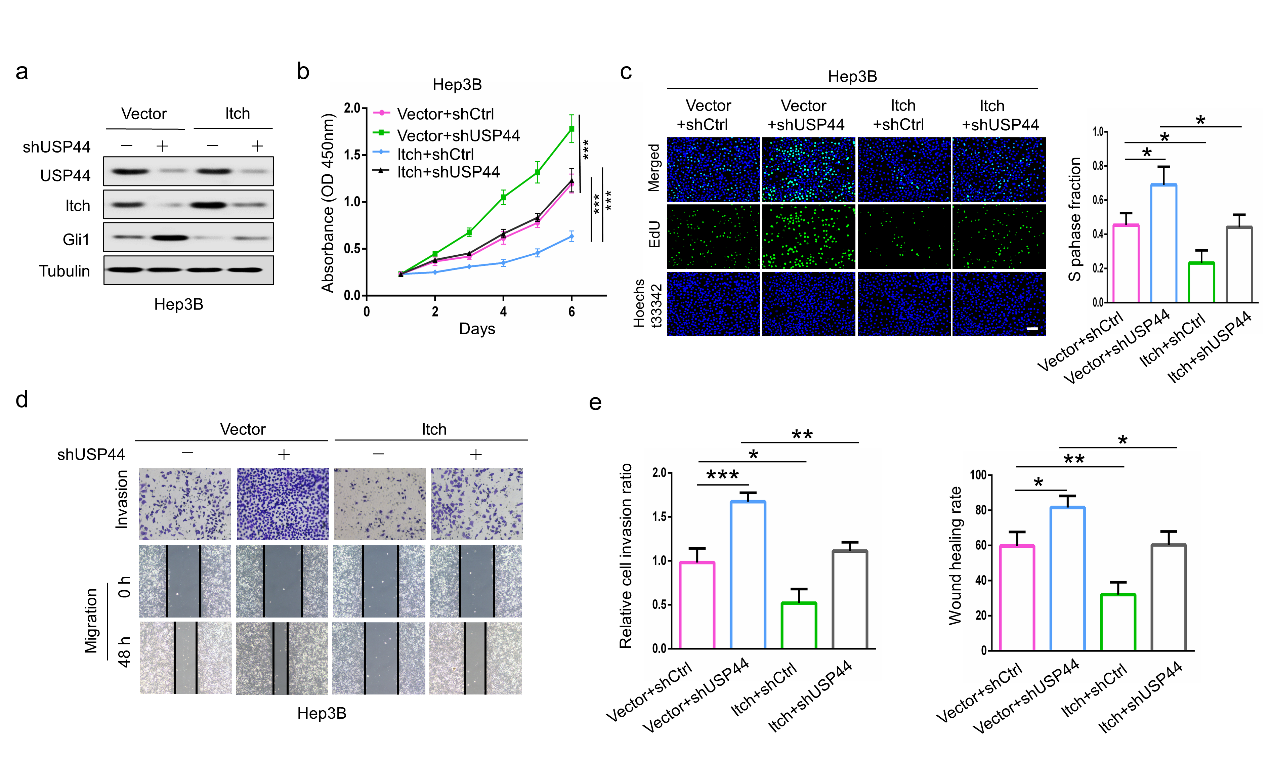
**

**Supplementary Fig. 4 USP44 attenuates the proliferation and migration of HCC cells by inhibiting the Hh signaling pathway *in vitro***

**a** Itch and Gli1 protein expression in USP44 knockdown Hep3B cells, with or without Itch overexpression. **b, c** CCK-8 and EdU analyses for cell proliferation in USP44 knockdown Hep3B cells, with or without Itch overexpression. **d, e** Transwell and wound healing assays were used to test cellular invasion and migration in USP44 knockdown Hep3B cells, with or without Itch overexpression. Data are obtained from three independent biological replicates and are presented as mean±SD. *p <0.05, **p <0.01, ***p <0.001 as indicated.


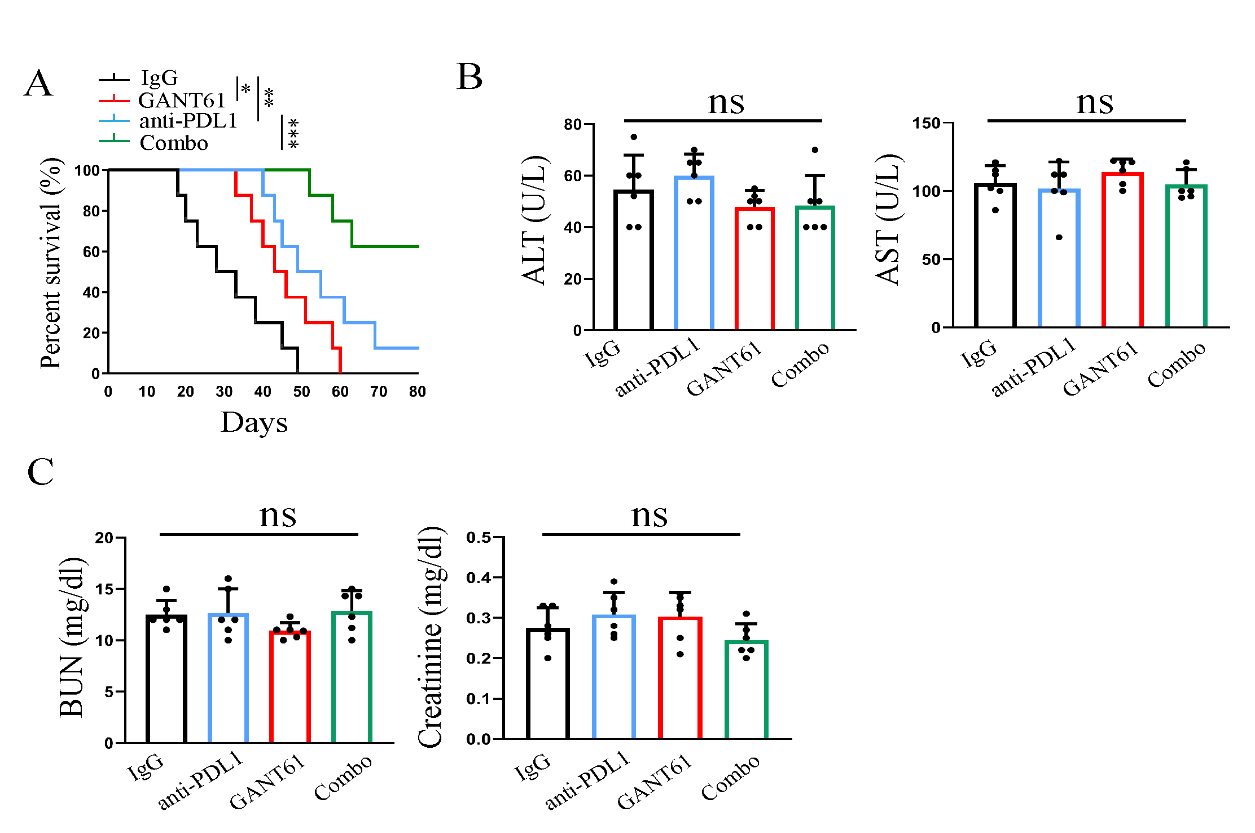


**Supplementary Fig. 5 Survival curves and serum indicators of liver and kidney function among the different groups of mice during treatment**

**a** Survival curves of mice bearing Hepa1-6 tumors that received GANT61, anti-PDL1 mAb or combination therapy. **b and c** Quantitative analysis of indicated biochemistry indices for liver and kidney function after the *in vivo* experiments. *p <0.05, **p <0.01, ***p <0.001 as indicated.
