# Supplementary material for: The deubiquitinating enzyme USP44 suppresses hepatocellular carcinoma progression by inhibiting Hedgehog signaling and PDL1 expression
Source: Cell Death Dis. 2023 Dec 14;14(12):830. doi: 10.1038/s41419-023-06358-y (PMC10721641; doi:10.1038/s41419-023-06358-y)
Supplement: Supplementary file 4 — Supplementary Materials and Methods [file 41419_2023_6358_MOESM4_ESM.doc]

**Supplementary Materials and Methods**

**Cell culture**

The human HCC cell lines (PLC, Huh7, Hep3B, HCCLM3, MHCC97H and HepG2), nontumoral cell line L02 and Hepa1-6 were obtained from the Cell Bank of Type Culture Collection of the Chinese Academy of Sciences (Shanghai, China). The cells were cultured in the DMEM media (Gibco, Grand Island, NY, USA) supplemented with 10% foetal bovine serum (Gibco, Grand Island, NY, USA), in humidified air containing 5% CO_2_ at 37°C.

**Constructs, transfection and reagents**

Overexpression USP44 plasmid (Flag-USP44) was constructed through inserting a PCR-amplified full-length fragment into the pCMV-Flag vector (Sigma-Aldrich，St. Loui, USA). The USP44 mutant construct (C282A) and Gli1 mutagenesis was down using the Stratagene QuikChange XL site-directed mutagenesis kit (Agilent, Santa Clara, USA). The lentiviral-based short hairpin RNAs (shRNAs) or overexpression plasmids used to silence USP44 gene were purchased from GenePharma (Shanghai, China). USP44 shRNA sequences #1 and #2 were obtained according to the shRNA sequence prediction website Portals. The shRNAs were synthesized and cloned into the pGMLV vector to obtain pGMLV-shUSP44 #1/2 plasmids. HCC stable cell lines were constructed through transfection of lentivirus containing indicated short hairpin RNAs (shRNAs). Puromycin (Invitrogen, Carlsbad, USA) was employed more than three weeks for selection. The shRNA sequences were provided as follows: shUSP44#1: 5’-GAGTATCAAGTTAAAGCAGAA-3’; shUSP44#2: 5’-ACTGAGAATGGACATTCTAAA-3’.

1‐stearoyl‐2‐arachidonoyl‐*sn*‐glycerol (SAG, SMO agonist) was supplied by Cayman Chemicals (Ann Arbor, MI, USA); MG132 and cyclohexamide (CHX) were purchased from Sigma–Aldrich.

**Western blot, Co-immunoprecipitation (Co-IP) and antibodies**

Western blot and Co-IP were performed according to our previous study [1]. Antibodies against USP44 (Santa Cruz, sc-377203, dilution 1:1000), Itch (Abcam, ab220637, dilution 1:1000), Gli1(Abcam, ab134906, dilution 1:1000), Gli2 (Santa Cruz, sc-271786, dilution 1:500), Gli3(Abcam, ab181130, dilution 1:1000), βTrCP (Santa Cruz, sc-390629, dilution 1:1000), pCAF (Santa Cruz, sc-13124, dilution 1:500), Skp2 (Thermo Fisher Scientific, 32-3300, dilution 1:500), SPOP (Proteintech, 16750-1-AP, dilution 1:1000), LATS1/2 (Proteintech, 202761-AP, dilution 1:1000), smad7(Abcam, ab216428, dilution 1:1000), RASSF5 (Santa Cruz, sc-80017, dilution 1:1000), WBP2 (Proteintech, 66585-1-lg, dilution 1:1000), PDL1 (Abcam, ab205921, dilution 1:1000), FLAG (Sigma, F1804, dilution 1:1000), Myc (Santa Cruz, sc-40, dilution 1:500), His (Proteintech, 66005-1-Ig, dilution 1:1000), GST (Santa Cruz, sc-53909, dilution 1:1000), HA (Abcam, ab1424, dilution 1:1000), Tubulin (Abcam, ab6160, dilution 1:2000) were utilized.

**quantitative real-time PCR and primers**

quantitative real-time PCR was performed as previously described [1]. Total RNA was extracted from Trizol reagent (Invitrogen, USA). Complementary DNA (cDNA) was prepared employing the PrimeScript RT Reagent Kit (Invitrogen, USA). qRT-PCR was performed using SYBR Premix Ex Taq (TaKaRa Bio, Shiga, Japan), following instructions of the manufacturer. Primers of genes were listed as follows:

USP44 Forward: CAGGACTAAGTGGAGCA；

USP44 Reverse: CCACGAAAGGCAGGAATGAG；

Itch Forward: TCACTTGGGCATAGGTCTCT；

Itch Reverse: TGTGCCCAGACACTGAGTTA；

Gli1 Forward: CTATGGTGAGCCATGCTGTC；

Gli1 Reverse: GAAAGTCCTTCTGTTCCCATGC；

Gli2 Forward: CCCCTACCGATTGACATGCG；

Gli2 Reverse: GAAAGCCGGATCAAGGAGATG；

Gli3 Forward: TGGTTACATGGAGCCCCACTA；

Gli3 Reverse: GAATCGGAGATGGATCGTAATGG；

Bcl2 Forward: CTGAGT ACCTGAACCGGCACC；

Bcl2 Reverse: GAGCAGAGTCTTCAGAGACAG；

c-Myc Forward: AACACACAACGTCTTGGAGCGCCA；

c-Myc Reverse: TCCTCTGCTTGGACGGACAGGATG；

Nanog Forward: CATCCTGAACCTCAGCTACAAACA；

Nanog Reverse: TTGCTATTCTTCGGCCAGTTGT；

FOXS1 Forward: GCTACATCATGGGCCGATTC；

FOXS1 Reverse: CAAACATGTCGTGGCAGTCA；

PDL1 Forward: GCTGCACTAATTGTCTATTGGGA；

PDL1 Reverse: AATTCGCTTGTAGTCGGCACC；

GAPDH Forward: GGAGCGAGATCCCTCCAAAAT；

GAPDH Reverse: GGCTGTTGTCATACTTCTCATGG.

**GST pull-down assay**

Interaction between USP44 and Itch was detected by GST-mediated pull-down assays (Thermo Scientific, Rockford, IL). HCCLM3 cells extracts with USP44 overexpression were obtained using pull-down lysis buffer (Thermo Scientific). Recombinant GST-Itch protein was expressed and purified. Total cell lysates with Flag-USP44 were incubated with purified GST-Itch linked to glutathione resin overnight at 4°C. After being washed with assay buffer, the eluted proteins were identified through western blot.

**Luciferase reporter assay**

The Hh-dependent luciferase experiment was carried out using HCC cells stably expressing a Gli-responsive luciferase reporter and the pRL-TK Renilla (normalization control). Dual-Luciferase Reporter Assay System (Promega) was conducted to detect luciferase and Renilla activities. All steps were carried out in strict accordance with the instructions.

***In vitro* deubiquitination assay**

Myc-Itch and HA-Ub were transfected into HEK293T cells. Then, ubiquitinated Itch was purified from the cell lysis with anti-Myc Sepharose in Myc-lysis buffer after 24h. When performing *in vitro* deubiquitination assay, recombinant USP44 and ubiquitinated Itch protein were incubated together for 2 h at 37°C in a deubiquitination buffer. The buffer contains 50 mmol/L Tris-HCl, 5 mmol/L MgCl_2_, 2 mmol/L DTT, and 2 mmol/L ATP-Na2 with proteasome inhibitors.

**Tandem Mass Tag™ (TMT) quantitative proteomics analysis**

USP44-knockdown Hep3B cell line was used to perform TMT quantitative proteomics analysis compared with the control group. Cells were lysed at 4 °C with urea lysis buffer supplemented with protease inhibitors and phosphatase inhibitors. Electrophoresis was used to separate extracted proteins on 12% SDS–PAGE at 220 V for 40 min. Proteins were labeled with TMT following the manufacturer’s procedure. Combined TMT samples were fractionated off-line into 24 fractions using high-pH reversed phase chromatography. For analysis of the global proteome, 500 ng peptides of each fraction were analyzed with LC–MS/MS. The remaining material was pooled into 12 fractions for each plex and subjected to phosphopeptide enrichment with immobilized metal affinity chromatography (IMAC) automated on an AssayMap Bravo System (Agilent) [2].

**References:**

1. Zhu H, Li Q, Zhao Y, Peng H, Guo L, Zhu J*, et al.* Vaccinia-related kinase 2 drives pancreatic cancer progression by protecting Plk1 from Chfr-mediated degradation. *ONCOGENE* 2021 2021-07-01; **40** (28)**:** 4663-4674.

2. Mertins P, Qiao JW, Patel J, Udeshi ND, Clauser KR, Mani DR*, et al.* Integrated proteomic analysis of post-translational modifications by serial enrichment. *NAT METHODS* 2013 2013-07-01; **10** (7)**:** 634-637.
