# Supplementary material for: The deubiquitinating enzyme USP44 suppresses hepatocellular carcinoma progression by inhibiting Hedgehog signaling and PDL1 expression
Source: Cell Death Dis. 2023 Dec 14;14(12):830. doi: 10.1038/s41419-023-06358-y (PMC10721641; doi:10.1038/s41419-023-06358-y)
Supplement: Supplementary file 5 — Table S1 [file 41419_2023_6358_MOESM5_ESM.docx]

| **Table S1-**Correlation between HCC USP44 expression and clinicopathologic features of patients enrolled. | | | | | | | | |  |  |
| --- | --- | --- | --- | --- | --- | --- | --- | --- | --- | --- |
|  |  |  |  |  |  |  |  |  |  |  |
| **Characteristics** |  | **Tumor USP44 expression** | | | | | ***P* value** |  |  |  |
|  |  |  | **Low (n=135)** |  | **High(n=56)** |  |  |  |  |  |
| Age (y) |  |  |  |  |  |  |  |  |  |  |
| ≤50 |  |  | 38 |  | 21 |  | 0.203 |  |  |  |
| >50 |  |  | 97 |  | 35 |  |  |  |  |  |
| Gender |  |  |  |  |  |  |  |  |  |  |
| Female |  |  | 45 |  | 19 |  | 0.937 |  |  |  |
| Male |  |  | 90 |  | 37 |  |  |  |  |  |
| HBsAg |  |  |  |  |  |  |  |  |  |  |
| Negative |  |  | 31 |  | 16 |  | 0.413 |  |  |  |
| Positive |  |  | 104 |  | 40 |  |  |  |  |  |
| HCVAg |  |  |  |  |  |  |  |  |  |  |
| Negative |  |  | 116 |  | 49 |  | 0.773 |  |  |  |
| Positive |  |  | 19 |  | 7 |  |  |  |  |  |
| ALT (U/L) |  |  |  |  |  |  |  |  |  |  |
| ≤50 |  |  | 89 |  | 39 |  | 0.619 |  |  |  |
| >50 |  |  | 46 |  | 17 |  |  |  |  |  |
| ALB (g/L) |  |  |  |  |  |  |  |  |  |  |
| ≤35 |  |  | 15 |  | 11 |  | 0.118 |  |  |  |
| >35 |  |  | 120 |  | 45 |  |  |  |  |  |
| AFP (ng/ml) |  |  |  |  |  |  |  |  |  |  |
| ≤400 |  |  | 112 |  | 43 |  | 0.321 |  |  |  |
| >400 |  |  | 23 |  | 13 |  |  |  |  |  |
| Tumor encapsulation |  |  |  |  |  |  |  |  |  |  |
| Complete |  |  | 113 |  | 46 |  | 0.793 |  |  |  |
| None |  |  | 22 |  | 10 |  |  |  |  |  |
| Tumor differentiation |  |  |  |  |  |  |  |  |  |  |
| I-II |  |  | 115 |  | 47 |  | 0.826 |  |  |  |
| III-IV |  |  | 20 |  | 9 |  |  |  |  |  |
| Tumor size (cm) |  |  |  |  |  |  |  |  |  |  |
| ≤5 |  |  | 85 |  | 45 |  | 0.019 |  |  |  |
| >5 |  |  | 50 |  | 11 |  |  |  |  |  |
| Tumor number |  |  |  |  |  |  |  |  |  |  |
| Single |  |  | 75 |  | 33 |  | 0.669 |  |  |  |
| Multiple |  |  | 60 |  | 23 |  |  |  |  |  |
| Distant metastasis |  |  |  |  |  |  |  |  |  |  |
| No |  |  | 109 |  | 53 |  | 0.015 |  |  |  |
| Yes |  |  | 26 |  | 3 |  |  |  |  |  |
| TNM stage |  |  |  |  |  |  |  |  |  |  |
| I-II |  |  | 103 |  | 50 |  | 0.041 |  |  |  |
| III-IV |  |  | 32 |  | 6 |  |  |  |  |  |
| *P* values were calculated using the Pearson chi-square test. *P* value of <0.05 was considered statistically significant. | | | | | | | | | |  |
|  |  |  |  |  |  |  |  |  |  |  |
| **Abbreviations:** HbsAg, hepatitis B surface antigen; ALT, alanine aminotransferase; ALB, albumin; AFP, alpha-fetoprotein; TNM stage, tumor node metastasis stage. | | | | | | | | | |  |
|  |  |  |  |  |  |  |  |  |  |  |
