# Supplementary material for: The deubiquitinating enzyme USP44 suppresses hepatocellular carcinoma progression by inhibiting Hedgehog signaling and PDL1 expression
Source: Cell Death Dis. 2023 Dec 14;14(12):830. doi: 10.1038/s41419-023-06358-y (PMC10721641; doi:10.1038/s41419-023-06358-y)
Supplement: Supplementary file 6 — Table S2 [file 41419_2023_6358_MOESM6_ESM.docx]

| **Table S2**-Univirate and multivariate analysis of factors associated with overall survival and recurrence free survival. | | | | | | | | | | | | |  |
| --- | --- | --- | --- | --- | --- | --- | --- | --- | --- | --- | --- | --- | --- |
| **Variables** | **OS** | | | | |  | **RFS** | | | |  | |  |
|  | **Univariate** | |  | **Multivariate** | |  | **Univariate** |  | **Multivariate** | |  |  |  |
|  | ***P*** | | ***P*** |  | **HR (95% CI)** | | ***P*** | ***P*** |  | **HR (95% CI)** | |  |  |
| Age (>50 years versus ≤50 years) | 0.635 |  | *NA* |  |  |  | 0.461 | *NA* |  |  |  |  |  |
| Gender (male versus female) | 0.316 |  | *NA* |  |  |  | 0.298 | *NA* |  |  |  |  |  |
| HbsAg (negative versus positive) | 0.139 |  | *NA* |  |  |  | 0.362 | *NA* |  |  |  |  |  |
| ALT (>50 versus ≤50) | 0.416 |  | *NA* |  |  |  | 0.523 | *NA* |  |  |  |  |  |
| ALB (>35 versus ≤35) | 0.517 |  | *NA* |  |  |  | 0.375 | *NA* |  |  |  |  |  |
| AFP (>400 versus ≤400) | **0.001** |  | **0.011** | **1.623 (1.302-2.263)** | | | **0.016** | **0.031** | **1.212 (1.112-2.327)** | | |  |  |
| Tumor encapsulation (none versus complete) | **0.003** |  | 0.526 | **1.332 (0.921-1.695)** | | | **0.001** | **0.017** | **1.533 (1.267-1.869)** | | |  |  |
| Tumor differentiation (III-IV versus I-II) | **0.036** |  | 0.386 | **1.212 (0.636-1.781)** | | | 0.614 | *NA* |  |  |  |  |  |
| Tumor size (>5 versus ≤5) | **0.001** |  | **0.033** | **1.397 (1.142-2.102)** | | | **<0.001** | **<0.001** | **1.851 (1.632-3.562)** | | |  |  |
| Tumor number (multiple versus single) | 0.212 |  | *NA* |  | | | 0.211 | *NA* |  |  |  |  |  |
| Distant metastasis (Yes versus No) | **<0.001** |  | **0.019** | **1.503 (1.158-2.518)** | | | 0.189 | *NA* |  | | |  |  |
| TNM stage (III-IV versus I-II) | **<0.001** |  | **0.228** | **1.561 (0.766-2.339)** | | | **0.327** | *NA* |  | | |  |  |
| USP44 expression (low versus high) | **<0.001** |  | **<0.001** | **2.106 (1.738-2.852)** | | | **<0.001** | **<0.001** | **1.822(1.616-2.573)** | | |  |  |
| Data obtained from the Cox proportional hazards model, *P*-value <0.05 was regarded as statistically significant. | | | | | | | | | | | | |  |
| **Abbreviations:** HbsAg, hepatitis B surface antigen; ALT, alanine aminotransferase; ALB, albumin; AFP, alpha-fetoprotein; TNM stage, tumor node metastasis stage. | | | | | | | | | | | | |  |
|  |  |  |  |  |  |  |  |  |  |  |  |  |  |
